# Structural insights into the gating mechanism of the fission yeast phosphate exporter SpXpr1

**DOI:** 10.1038/s41421-026-00883-8

**Published:** 2026-04-15

**Authors:** Hui Yang, Yuechan Wang, Chenxi Yue, Xinran Li, Yifei Wang, Ye Yu, Huaizong Shen

**Affiliations:** 1 College of Life Sciences, Zhejiang University, Hangzhou, Zhejiang China; 2 Zhejiang Key Laboratory of Structural Biology, School of Life Sciences, Westlake University, Hangzhou, Zhejiang China; 3 Westlake Laboratory of Life Sciences and Biomedicine, Hangzhou, Zhejiang China; 4 Westlake Institute for Advanced Study, Hangzhou, Zhejiang China; 5 School of Basic Medicine and Clinical Pharmacy and State Key Laboratory of Natural Medicines, China Pharmaceutical University, Nanjing, Jiangsu China

**Keywords:** Electron microscopy, Protein transport

## Abstract

Phosphate homeostasis is essential for fundamental cellular processes, including energy metabolism, signal transduction, and nucleic acid synthesis. Although XPR1 family proteins are conserved phosphate exporters throughout eukaryotes, their structural mechanisms in organisms other than mammals and plants remain largely unexplored. Here, we presented high-resolution cryo-electron microscopy (cryo-EM) structures of *Schizosaccharomyces pombe* Xpr1 (SpXpr1) in both the apo and inositol hexakisphosphate (InsP6)-bound states. While SpXpr1 shares conserved phosphate coordination sites with its human and plant orthologs, SpXpr1 employs a unique dual gating mechanism: (1) an intracellular gate formed by the N-loop of the SPX domain, stabilized by a preceding N-helix and an extended TM10 helix and (2) an extracellular ECL_plug_ occluding the exit. We further showed that InsP6 binding induces allosteric destabilization of the N-loop gate, facilitating phosphate release. Functional validation through phosphate efflux assays in *Homo sapiens* *XPR1* (*HsXPR1*)-knockout cells and whole-cell patch-clamp recordings confirmed the structural observations. Our findings elucidated a unique gating mechanism of SpXpr1 and offer evolutionary perspectives on phosphate regulation across eukaryotes.

## Introduction

Phosphorus is an essential macronutrient that underpins life, serves as a critical component of ATP, nucleic acids, phospholipids, and signaling molecules (e.g., phosphorylated proteins) and contributes to structural biomineralization in vertebrates^1–5^. Within cells, inorganic phosphate (Pi) acts as the primary biological phosphorus carrier, playing additional roles in buffering the pH and regulating enzymatic activity^6–8^. Tight control of Pi homeostasis is therefore indispensable across all organisms^2,7^.

In *Schizosaccharomyces pombe*, Pi balance is maintained through the coordinated interplay of transport, storage, and metabolic pathways^7,9^. Under Pi starvation, cells induce the expression of the acid phosphatase Pho1 to scavenge extracellular organic phosphates and upregulate high-affinity transporters (Pho84 and Pho842) for Pi uptake^10–12^. Conversely, under Pi-replete conditions, excess uptake is suppressed via the Pqr1/Spx1-mediated degradation of these transporters, while the VTC complex sequesters excess Pi as polyphosphate^7,12^. Intriguingly, genetic evidence points to Xpr1/Spx2 as a critical Pi exporter: its activity is markedly enhanced in *Pqr1* and *VTC* double-knockout (KO) strains, suggesting that efflux plays a key role in preventing cytosolic Pi overload^7^.

XPR1 family proteins exhibit remarkable functional diversification across eukaryotes^3,7,13,14^. In *Saccharomyces cerevisiae*, Syg1 regulates G protein signaling but lacks demonstrated Pi transport activity^15^. Plant PHO1 mediates root-to-shoot Pi translocation under stringent control through ubiquitination and transcriptional networks^14^. In humans, mutations in XPR1/SLC53A1 — the only known Pi exporter — are linked to pathologies such as brain calcification and renal dysfunction^13,16–20^. Despite these divergent roles, all XPR1 homologs share a conserved architecture: an N-terminal SPX domain that senses inositol polyphosphates (IPs, e.g., InsP6–InsP8) and a C-terminal EXS domain (named after Erd1, XPR1, and Syg1), which is embedded within the 10-helix transmembrane core and is responsible for Pi transport^13,21–27^. This modular design enables cells to couple intracellular Pi levels with export activity through IP-dependent allosteric regulation^21,24,25,28^. Although SPX domains have been structurally characterized and recent studies have revealed the transport mechanisms of human XPR1 and plant PHO1, the principles governing phosphate export in evolutionarily distant species remain poorly understood^3,29–42^.

Here, we report high-resolution cryo-electron microscopy (cryo-EM) structures of *S. pombe* Xpr1 (SpXpr1) in both the apo and InsP6-bound states. Combining structural analysis with electrophysiological characterization and phosphate efflux assays in a clean genetic background, we reveal a Pi transport mechanism in fission yeasts that diverges fundamentally from that of its human counterpart. Our findings provide evolutionary insights into phosphate export across eukaryotes.

## Results

### Functional characterization of SpXpr1

**Fig. 1:**
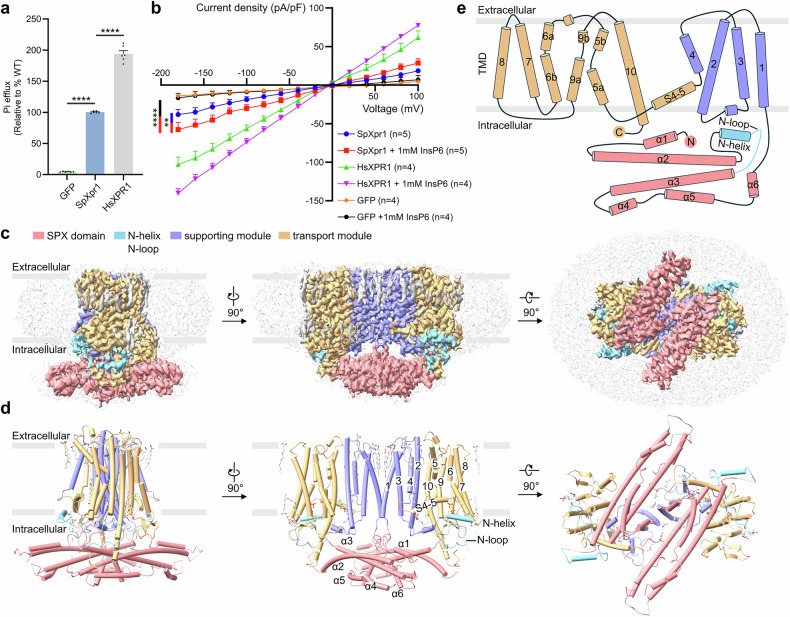
Functional characterization and structural determination of SpXpr1. **a** Phosphate export activity of SpXpr1 and HsXPR1 in the *HsXPR1*-KO 293T cell line. The efflux activity of each group was normalized to its protein expression level as determined by western blotting and then compared to that of SpXpr1 (set to 100%). GFP-expressing cells served as negative controls. The results are presented as the mean ± SEM, with *n* = 6 from three independent experiments. Statistical analysis was performed using one-way ANOVA with Dunnett’s test for comparisons with the wild-type (WT) group. **b** Current‒voltage (*I*‒*V*) relationship of phosphate permeation in cells expressing SpXpr1, HsXPR1, or GFP. The data were analyzed by two-way ANOVA with Tukey’s test (SpXpr1 + InsP6 vs GFP + InsP6: *P* < 0.0001; SpXpr1 + InsP6 vs SpXpr1: *P* = 0.0077). Significance levels: *P ≤ 0.05, **P ≤ 0.01, ***P ≤ 0.001, ****P ≤ 0.0001; ns, not significant. **c** Three representative views of the cryo-EM density map of SpXpr1 colored by structural elements: SPX domain (pink), N-helix and N-loop (sky blue), supporting module (purple; TM1–4), transport module (yellow; TM5–10), and bound lipids (light gray). All structural maps and models presented in this study were generated using ChimeraX^51^. **d** Cartoon representation of SpXpr1 in an orientation matched to that in **c**, highlighting domain organization and key structural features. **e** Topological map showing the membrane organization of SpXpr1, with numbered transmembrane helices (TM1–10) and cytoplasmic domains.

To assess the phosphate export activity of SpXpr1 (UniProt: Q9UU86), we generated a stable *Homo sapiens* *XPR1* (*HsXPR1*)-KO HEK293T cell line to eliminate endogenous background interference (Supplementary Fig. S1). We transiently expressed SpXpr1 and HsXPR1 in these KO cells, verified their expression and subcellular localization using fluorescence microscopy (Supplementary Fig. S2), and measured extracellular phosphate accumulation. To ensure a rigorous comparison, efflux rates were normalized to protein expression levels quantified via western blotting (Supplementary Fig. S3). Compared with GFP-expressing controls, SpXpr1-expressing cells exhibited a robust time-dependent increase in phosphate efflux, although with a lower efficiency than that of human XPR1, which is consistent with the findings of previous functional studies (Fig. 1a).

Recent studies have established that HsXPR1 functions as a phosphate channel, which has been validated through electrophysiological analysis. To determine whether this transport mechanism is conserved in fungal orthologs, we performed whole-cell patch-clamp recordings. In contrast to the GFP control cells, SpXpr1-expressing cells exhibited significant Pi-dependent currents across a range of membrane potentials, indicating direct Pi conductance (Fig. 1b). Moreover, similar to its human ortholog, the channel activity of SpXpr1 was potentiated by the addition of InsP6, supporting its regulation by inositol phosphates (Fig. 1b). These electrophysiological data establish SpXpr1 as a bona fide phosphate channel.

### Overall architecture of SpXpr1

For structural analysis, we expressed full-length SpXpr1 with an N-terminal FLAG tag in HEK293F cells and purified it via affinity and size-exclusion chromatography (SEC). The apo-state structure, obtained from protein purified in phosphate buffer (10 mM NaH_2_PO_4_/Na_2_HPO_4_), was resolved by cryo-EM at 2.90 Å resolution (Fig. 1c, d; Supplementary Figs. S4–S7 and Table S1). To investigate IP-mediated regulation, we also determined the structure of SpXpr1 bound to InsP6 (SpXpr1^+InsP6^) at 2.87 Å resolution, which revealed a slight rotation of the SPX domain. Given the nearly identical overall conformations of the two states, we primarily describe the apo structure below, reserving discussion of the InsP6-bound conformation for analysis of IP-dependent activation mechanisms.

SpXpr1 forms a symmetric homodimer (Fig. 1c, d), mirroring its human ortholog^32–41^. Each protomer comprises an N-terminal cytosolic SPX domain, which folds into a six-helix bundle (α1–α6), and a transmembrane domain (TMD) with 10 helices (Fig. 1e; Supplementary Fig. S8). The TMD is organized into two functional modules: a supporting module (TM1–4) that stabilizes the dimer interface, aided by bound lipids (Supplementary Fig. S9a, b), and a transport module (TM5–10) that encloses the ion-conducting pathway. The two modules are connected by an interfacial helix (S4–5) that connects TM4 and TM5, with a bifurcated lipid molecule located between them (Supplementary Fig. S9c).

### Pi coordination along the transport pathway

**Fig. 2:**
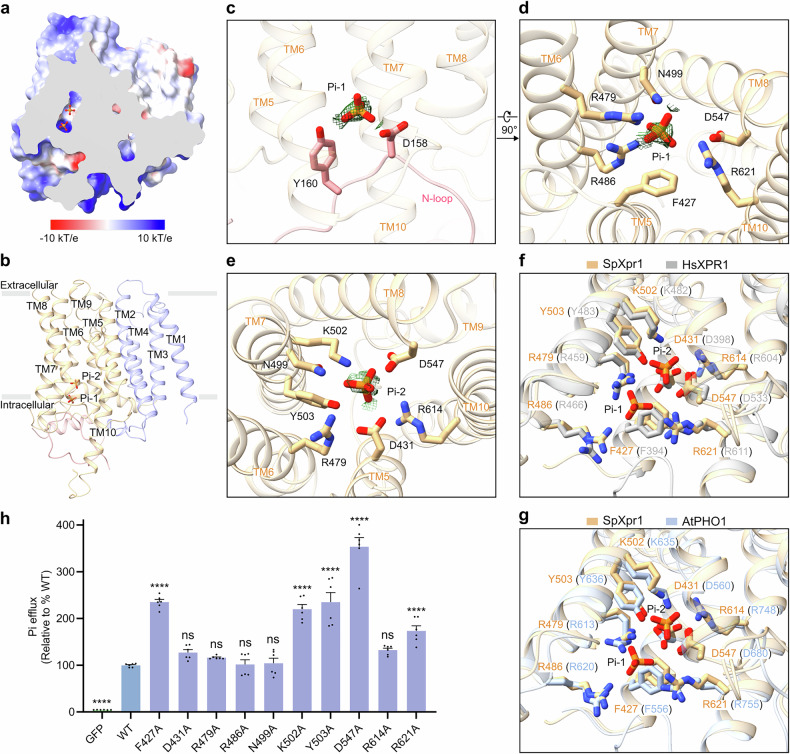
Phosphate coordination along the transport pathway. **a** Electrostatic surface potential of the SpXpr1 pore, shown in a cut-open slice view. Positive and negative electrostatic potentials are colored blue and red, respectively. **b** Structural localization of the two phosphate ions (Pi-1 and Pi-2) along the transport pathway of SpXpr1. **c**–**e** Detailed coordination mechanisms for Pi-1 (**c**, **d**) and Pi-2 (**e**), with key interacting residues and phosphate ions shown as stick models. Electron microscopy densities of Pi ions are shown as green meshes and contoured at 4*σ*. **f**, **g** Structural alignment of the phosphate-binding sites of SpXpr1 and HsXPR1 (Protein Data Bank (PDB): 8X5B)^32^ (**f**) and AtPHO1 (PDB: 9JF8)^42^ (**g**), demonstrating evolutionary conservation of coordination geometry. **h** Functional impact of Pi-coordinating residue mutations on phosphate export activity. The efflux activity of each group was normalized to its protein expression level as determined by western blotting and then compared to that of the WT (set to 100%). GFP-expressing cells served as negative controls. The results are presented as the mean ± SEM, with *n* = 6 from three independent experiments. Statistical analysis was performed using one-way ANOVA with Dunnett’s test for comparisons with the WT group (*P ≤ 0.05, **P ≤ 0.01, ***P ≤ 0.001, ****P ≤ 0.0001; ns not significant).

Examination of the SpXpr1 structure at a stringent contour level of 4*σ* revealed two distinct densities along the transport pathway, each surrounded by clusters of positively charged residues (Fig. 2a–e). We designated these sites as Pi-1 and Pi-2, which proceed from the cytosolic to the extracellular side. We propose that these discernible densities likely result from the ensemble averaging of dynamic transport states, representing stable intermediates within the channel. Both phosphate ions engage in extensive interactions with the protein: Pi-1 is positioned at the cytosolic entrance, coordinated by hydrogen bonds with D158, Y160 (N-loop), and N499 (TM7), in addition to electrostatic interactions with R479/R486 (TM6) and R621 (TM10) (Fig. 2c, d). Progressing extracellularly, Pi-2 is stabilized by a hydrophilic network involving D431 (TM5), R479 (TM6), N499/K502/Y503 (TM7), D547 (TM8), and R614 (TM10) (Fig. 2e).

Structural comparison with HsXPR1 and *Arabidopsis* PHO1 revealed that these coordination residues are highly conserved across kingdoms^32,42^ (Fig. 2f, g). Functional characterization via mutagenesis, normalized to protein expression levels (Supplementary Figs. S2, S3) or total cellular proteins (Supplementary Fig. S10), revealed divergent effects on transport efficiency (Fig. 2h). While some mutants (e.g., D431A) displayed minimal influence on efflux rates, others (e.g., F427A) unexpectedly exhibited significantly enhanced transport activity. This phenotype stands in sharp contrast to the impaired activity typically observed for corresponding mutations in HsXPR1. Although initially counterintuitive, we attribute this discrepancy to the unique gating mechanism of SpXpr1, as detailed in the subsequent section. We propose that these mutations likely facilitate efflux by destabilizing the auto-inhibited state adopted by the transporter in the absence of endogenous activators. Alternatively, the substitution of bulky side chains with alanine may result in a slightly enlarged pore diameter.

### Intracellular gating by the N-loop in the SPX domain

**Fig. 3:**
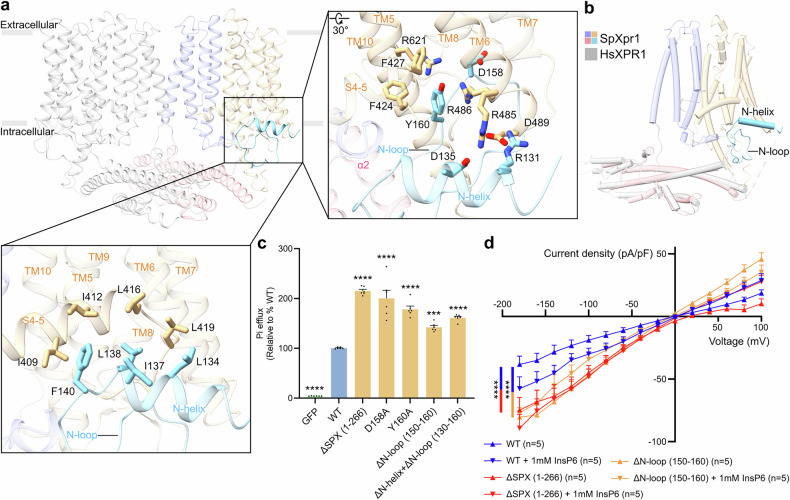
Structural and functional characterization of the N-loop gating mechanism in SpXpr1. **a** Structural characterization of the N-helix and N-loop in the SPX domain of SpXpr1, highlighting key molecular interactions, including hydrophilic and hydrophobic interactions. The relevant residues are depicted as stick models. **b** Comparative structural analysis of the SPX domains of SpXpr1 and HsXPR1 (PDB: 8X5F)^32^, revealing the absence of the N-helix and N-loop in HsXPR1. **c** Phosphate export activities of WT SpXpr1 and SpXpr1 mutants involved in N-loop stabilization. The efflux activity of each group was normalized to its protein expression level as determined by western blotting and then compared to that of the WT (set to 100%). Cells expressing GFP served as controls. The results are presented as the mean ± SEM, with *n* = 6 from three independent experiments. Statistical analysis was performed using one-way ANOVA with Dunnett’s test for comparisons with the WT group. **d** *I*‒*V* relationship of phosphate permeation in cells expressing WT SpXpr1 or the ΔSPX (1–266) or ΔN-loop (150–160) mutants. The data were analyzed by two-way ANOVA with Tukey’s test (WT vs ΔSPX (1–266): *P* < 0.0001; WT vs ΔN-loop (150–160): *P* < 0.0001; ΔSPX (1–266) vs ΔN-loop (150–160): *P* = 0.0064). Significance levels: *P ≤ 0.05, **P ≤ 0.01, ***P ≤ 0.001, ****P ≤ 0.0001; ns not significant.

A key structural distinction between SpXpr1 and HsXPR1 is the presence of a stable N-helix (residues 130–139) and N-loop (residues 140–161) between the α2 and α3 helices of the SPX domain^32^ (Figs. 1d, e, 3a, b; Supplementary Fig. S8). These elements engage in extensive interactions with the transport module (TM5–10): the N-helix participates in electrostatic interactions (R131–D489 and D135–R485 in TM6), while the N-loop mediates multiple contacts, including D158–R486 (in TM6), Y160–F427 (in TM5) (π–π), and Y160–R486 (in TM6)/R621 (in TM10) (cation–π) interactions (Fig. 3a). Additional stabilization comes from hydrophobic interactions between the C-terminus of the N-helix (L134, I137, L138, and F140) and the S4-5 linker (I409, I412, L416, and L419) (Fig. 3a).

Phylogenetic analysis of sequence and structural alignment data revealed that the N-helix/N-loop module is a conserved feature across the *Schizosaccharomyces* genus (Supplementary Fig. S11). The N-loop occupies a position structurally analogous to the C-terminal tail in HsXPR1, which is known to gate the intracellular entrance. We therefore propose that this network positions the N-loop as a gate that blocks intracellular Pi entry, with the N-helix reinforcing this occlusion. In support of this model, Pi efflux assays demonstrated that disrupting this gating mechanism — through N-loop deletion, SPX domain removal, or point mutations in critical residues — significantly increases transport activity (Fig. 3c). Consistent with these findings, electrophysiological recordings revealed that both the ΔSPX and Δ150–160 mutants exhibited higher basal phosphate conductance than the WT did (Fig. 3d).

### Divergent C-terminal gating mechanism in SpXpr1

**Fig. 4:**
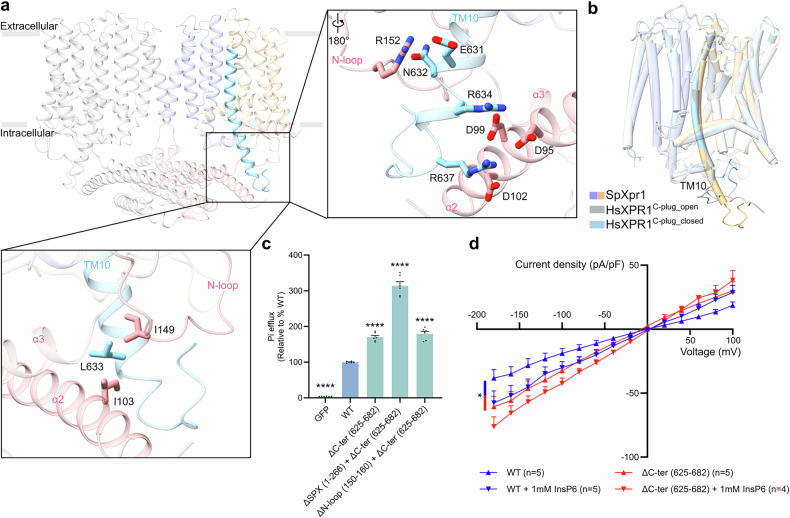
Distinct C-terminal architecture mediates SpXpr1 regulation. **a** The extended C-terminal helical structure of TM10 projects into the cytosol and forms both hydrophilic and hydrophobic interactions with the SPX domain. Relevant interactions are displayed as sticks. **b** Comparative structural analysis of the C-terminal structures of SpXpr1 and HsXPR1 in both the closed (PDB: 8X5B) and open (PDB: 8X5F) states^32^, emphasizing the unique helical extension of TM10 in SpXpr1. **c** Phosphate export activities of the WT and mutants involving the C-terminal region. The efflux activity of each group was normalized to its protein expression level as determined by western blotting and then compared to that of the WT (set to 100%). Cells expressing GFP served as controls. The results are presented as the mean ± SEM, with *n* = 6 from three independent experiments. Statistical analysis was performed using one-way ANOVA with Dunnett’s test for comparisons with the WT group. **d** *I*‒*V* relationship of phosphate permeation in cells expressing WT SpXpr1 or the ΔC-ter (625–682) mutant. The data were analyzed by two-way ANOVA with Tukey’s test (WT vs ΔC-ter (625–682): *P* = 0.0035). Significance levels: *P ≤ 0.05, **P ≤ 0.01, ***P ≤ 0.001, ****P ≤ 0.0001; ns not significant.

Unlike HsXPR1, SpXpr1 features an extended TM10 helix that projects into the cytosol to form extensive contacts with the SPX domain^32^ (Fig. 4a, b). Whereas HsXPR1 employs a C-terminal loop (C-plug) that sterically blocks the transport pathway in its apo state^32–41^, SpXpr1 leverages this elongated TM10 helix to rigidify the SPX domain, thereby stabilizing N-loop-mediated auto-inhibition. This architectural divergence likely originates from a glutamate substitution (E631) at the position corresponding to human G621 (Supplementary Fig. S8). The extended TM10 helix engages the SPX domain through multiple interactions: electrostatic interactions between TM10 residues E631, R634, and R637 and SPX domain residues D95, D99, D102, and R152, complemented by hydrophobic interactions between L633 (TM10) and I103/I149 (SPX domain) (Fig. 4a).

We propose that this interaction network stabilizes the SPX domain in a conformation that maintains channel closure, reinforcing the pore occlusion mediated by the N-loop. This auto-inhibition model is strongly supported by functional analysis: disrupting this stabilization via C-terminal truncation (residues 625–682) robustly increased transport activity compared with that of WT SpXpr1, resulting in significantly increased phosphate export in efflux assays and increased phosphate-conducting currents in whole-cell patch-clamp recording experiments (Fig. 4c, d).

### Extracellular gating by ECL_plug_

**Fig. 5:**
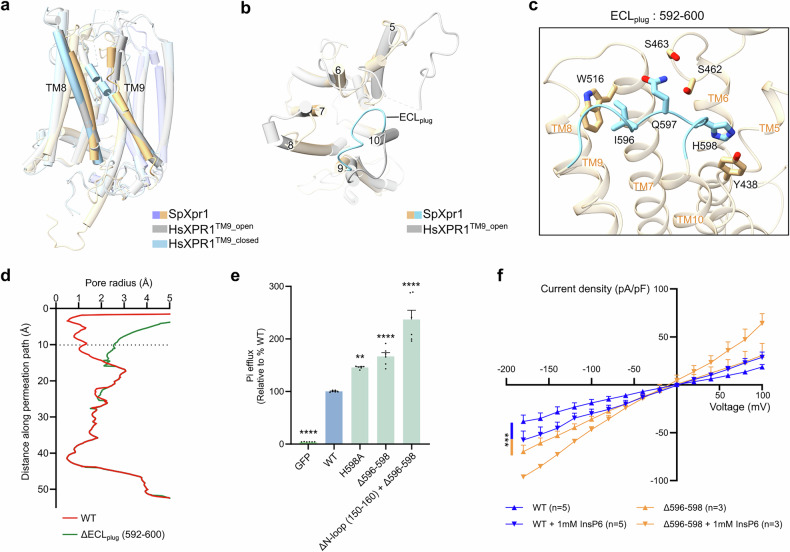
ECL_plug_ occludes the transport pathway from the extracellular side. **a** Structural comparison showing that SpXpr1 TM9 maintains a conformation similar to that of HsXPR1 in the open state (PDB: 9J98), which is distinct from the bent conformation of TM9 in closed-state HsXPR1 (PDB: 9J97)^36^. **b** Superposition reveals the unique TM9–TM10 loop of SpXpr1 (termed ECL_plug_, residues 592–600) that occludes the extracellular exit, which is absent in HsXPR1. **c** Molecular interactions stabilizing the ECL_plug_ are depicted as sticks. **d** HOLE^60^ analysis of ion conductance pathways confirms the physical occlusion of the pathway by ECL_plug_ and its alleviation in SpXpr1^Δ592–600^. **e** Functional analysis of the ECL_plug_ mutants revealed increased phosphate export with both the point mutant (H598A) and deletion mutant (Δ596–598) compared with that of the WT. The efflux activity of each group was normalized to its protein expression level as determined by western blotting and then compared to that of the WT (set to 100%). Cells expressing GFP served as controls. The results are presented as the mean ± SEM, with *n* = 6 from three independent experiments. Statistical analysis was performed using one-way ANOVA with Dunnett’s test for comparisons with the WT group. **f** *I*‒*V* relationship of phosphate permeation in cells expressing WT SpXpr1 or the Δ596–598 mutant. The data were analyzed by two-way ANOVA with Tukey’s test (WT vs Δ596–598: *P* = 0.0006). Significance levels: *P ≤ 0.05, **P ≤ 0.01, ***P ≤ 0.001, ****P ≤ 0.0001; ns not significant.

While human XPR1 regulates extracellular access through conformational changes in TM9, with its closed state bending toward the exit of the transport pathway^32–41^, SpXpr1 employs a distinct mechanism. The structures of both apo SpXpr1 and InsP6-bound SpXpr1 show that TM9 maintains a straight helical conformation resembling that of activated HsXPR1^36^ (Fig. 5a). Thus, extracellular occlusion is instead mediated by a loop between TM9 and TM10 (residues 592–600, termed ECL_plug_), which physically blocks transport pathway exit (Fig. 5b, c). ECL_plug_ stabilization involves three key interactions: (1) hydrophobic contacts with I596 and W516 (TM7), (2) hydrogen bonds linking Q597 to S462/S463 (TM5–TM6 loop), and (3) π–π stacking of H598 with Y438 (TM5) (Fig. 5c). HOLE analysis revealed a markedly enlarged pore radius at the extracellular exit in the absence of ECL_plug_, supporting its function as an extracellular gate (Fig. 5d).

This unique ECL_plug_ architecture is highly conserved across fission yeasts, underscoring its evolutionary importance (Supplementary Fig. S12). We propose that compared with human XPR1, ECL_plug_ may explain the lower transport activity of SpXpr1 (Fig. 1d, e). In support of this hypothesis, disruption of ECL_plug_ (via deletion or H598A mutation) significantly increased phosphate export (Fig. 5e). Consistent with these findings, whole-cell patch-clamp recordings confirmed that compared with WT SpXpr1, the deletion of ECL_plug_ resulted in greater phosphate conductance (Fig. 5f). Collectively, these functional data, complemented by structural modeling, establish ECL_plug_ as a critical physical constriction that gates the extracellular end of the transport pathway.

### Conformational changes induced by InsP6 binding

**Fig. 6:**
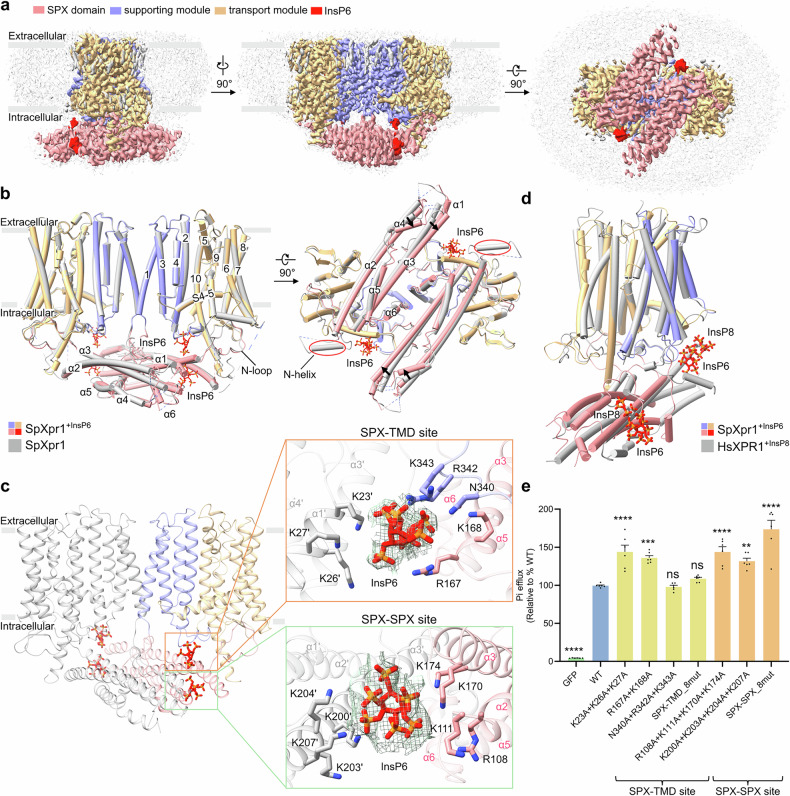
Conformational changes induced by InsP6 binding. **a** Cryo-EM density map of SpXpr1^+InsP6^ presented in three orthogonal views, colored by structural element: SPX domain (pink), supporting module (purple), transport module (yellow), InsP6 (red), and bound lipids (light gray). **b** Structural superposition of apo SpXpr1 and SpXpr1^+InsP6^ in two orientations reveals ligand-induced conformational changes, particularly in the SPX domain (rotation is indicated by black arrows). **c** Molecular details of InsP6 coordination at the SPX–TMD interface and SPX dimer interface, with interacting residues shown as sticks. Electron microscopy densities of InsP6 molecules are shown as green meshes and contoured at 8*σ*. **d** Comparative structural analysis of inositol polyphosphate binding geometries between SpXpr1^+InsP6^ and HsXPR1^+InsP8^ (PDB: 9DVP)^37^. **e** Phosphate export activities of the WT and mutants involved in InsP6 binding. The efflux activity of each group was normalized to its protein expression level as determined by western blotting and then compared to that of the WT (set to 100%). Cells expressing GFP served as controls. The results are presented as the mean ± SEM, with *n* = 6 from three independent experiments. Statistical analysis was performed using one-way ANOVA with Dunnett’s test for comparisons with the WT group (*P ≤ 0.05, **P ≤ 0.01, ***P ≤ 0.001, ****P ≤ 0.0001; ns not significant).

To investigate the mechanism of activation by IPs, we determined the structure of SpXpr1 bound to InsP6 (Fig. 6a, b; Supplementary Figs. S4–S6, S13 and Table S1). The SpXpr1^+InsP6^ structure has two InsP6 binding sites that are evolutionarily conserved with those of human XPR1^37^ (Fig. 6a–d). The first binding pocket, located at the SPX–TMD interface, coordinates InsP6 through electrostatic interactions with K23, K26, and K27 (α1 helix), R167 and K168 (adjacent SPX domain), and N340, R342, and K343 (TM2–TM3 loop) (Fig. 6c). The second binding site located at the SPX dimer interface similarly engages InsP6 through multiple basic residues (K200/K203/K204/K207 from one protomer and R108/K111/K170/K174 from the other) (Fig. 6c).

Comparative structural analysis revealed that while the TMD remains largely unchanged, InsP6 binding induces slight yet significant clockwise rotation of the SPX domain when viewed from the intracellular side (Fig. 6b). This rotation coincides with the complete destabilization of the regulatory N-helix, as evidenced by the loss of its cryo-EM density; however, the N-loop gate remains positioned to block intracellular entry (Fig. 6a, b). This limited structural rearrangement aligns with our functional observations, wherein InsP6 increases phosphate conductance in WT SpXpr1, supporting its role as a physiological modulator (Fig. 1b).

To assess the physiological relevance of these sites, we performed efflux assays with binding site mutants. Intriguingly, mutagenesis of the conserved InsP6 binding sites at both the SPX–TMD and SPX dimer interfaces resulted in minimal impairment of or even increased phosphate export (Fig. 6e). This stands in stark contrast to the severe functional loss caused by analogous mutations in human XPR1^32,33,37,38^. We attribute this divergence to the distinct gating mechanism of SpXpr1, wherein IP binding is functionally repurposed from being an obligatory trigger (as in HsXPR1) to acting as a fine-tuning modulator. Consequently, mutations at these sites may not disrupt an essential activation step but could perturb the auto-inhibitory lock, thereby lowering the energy barrier for activation-related conformational changes.

### Working model for SpXpr1-mediated phosphate efflux

**Fig. 7:**
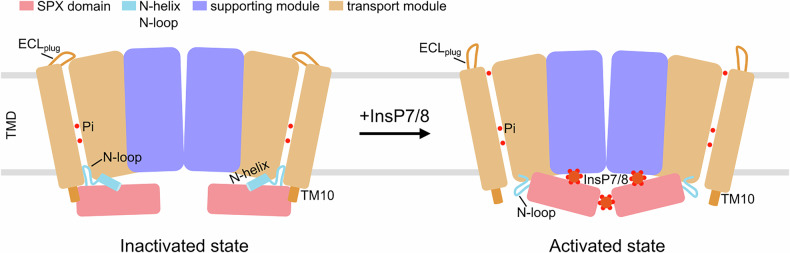
Proposed working model for Pi efflux by SpXpr1. In the apo/inactivated state, SpXpr1 is auto-inhibited by a dual-gating mechanism involving the intracellular N-loop and extracellular ECL_plug_. The binding of endogenous activators, likely InsP7 and InsP8, allosterically relieves this inhibition by inducing rotation of the dimeric SPX domain, which destabilizes the gating elements and facilitates phosphate release.

We integrated our findings into a working model for SpXpr1-mediated phosphate efflux (Fig. 7). In its resting state, SpXpr1 is auto-inhibited by a unique dual-gating mechanism involving the intracellular N-loop and extracellular ECL_plug_ to conserve intracellular phosphate. Efflux is initiated when elevated levels of intracellular phosphate lead to the synthesis and binding of high-energy inositol pyrophosphates (e.g., InsP7/InsP8). Binding of these activators induces rotation of the SPX domain, which subsequently displaces the extended TM10 helix and destabilizes the stabilizing N-helix, in turn releasing the inhibitory N-loop gate and ECL_plug_ to facilitate phosphate efflux.

## Discussion

Our structural and functional analyses revealed that SpXpr1 employs a unique dual-gating mechanism for phosphate export that diverges fundamentally from its human ortholog. While HsXPR1 utilizes a dynamic C-plug to regulate transport^32–41^, SpXpr1 features a distinct mechanism: (1) an intracellular gate formed by the N-loop of the SPX domain, stabilized by a preceding N-helix and (2) an extracellular ECL_plug_ (TM9–TM10 loop) that occludes the exit pathway. This mechanistic divergence is underscored by sequence and functional differences; unlike HsXPR1, where SPX domain truncation decreases activity^32,36,37^, the removal of the SPX domain in SpXpr1 increases transport, indicating its primary role in stabilizing the inhibitory N-loop rather than facilitating activation.

The structural basis for these functional differences becomes more evident when the C-terminal architectures of SpXpr1 and HsXPR1 are compared^32–41^. In SpXpr1, TM10 extends into the cytosol as an elongated helix that interacts extensively with the SPX domain, whereas the C-terminus of HsXPR1 forms a flexible loop that directly plugs the intracellular entrance. This fundamental architectural difference underlies their distinct regulatory strategies.

Notably, our structural data suggest that InsP6 may be insufficient for full activation of SpXpr1. The limited rotation of the SPX domain induced by InsP6 binding fails to completely displace the inhibitory N-loop, implying that more highly charged inositol pyrophosphates such as InsP7 and InsP8^3,12,25,43^, owing to their additional negative charges, may serve as the physiological activators capable of inducing the substantial conformational changes required for channel opening.

Our findings support the model proposed by Takado et al. in which SpXpr1 functions as part of an integrated phosphate regulatory network in fission yeasts, working in concert with vacuolar phosphate storage systems such as the VTC complex^7,12^. This coordination is evidenced by the synthetic lethality observed in Δ*pqr1*Δ*xpr1*Δ*vtc4* strains^7^, suggesting that SpXpr1 provides a critical backup mechanism when vacuolar phosphate storage is compromised. The distinct activation thresholds and regulatory mechanisms between fungal and mammalian XPR1 orthologs may reflect their different physiological contexts, wherein human cells require the capability for rapid phosphate export and yeast cells prioritize stringent conservation of phosphate stores^7,10,29^.

There are several limitations to the current study. First, our functional characterization of SpXpr1 and its mutants was performed in a heterologous mammalian expression system (HEK293T cells). While this approach allowed for controlled efflux assays and demonstrated that the core gating mechanism is operational outside the native context, future validation within *S. pombe* will be essential to confirm its physiological regulation. Second, it remains to be definitively established whether the functional impact of mutations in critical phosphate-coordinating or inositol phosphate-binding residues is mediated specifically through the unique N-loop gate. A combinatorial genetic approach, such as pairing these point mutations with deletion of the N-loop, will be a priority for future work to dissect this allosteric pathway. Finally, to fully elucidate the activation mechanism, a high-resolution structure of SpXpr1 in an activated state, ideally in complex with a high-affinity inositol pyrophosphate ligand such as InsP7 or InsP8, is needed.

These findings significantly advance our understanding of phosphate transport regulation across eukaryotes. The mechanistic difference between SpXpr1 and HsXPR1 highlights the evolutionary plasticity of the XPR1 family while preserving its core function in phosphate homeostasis. From an applied perspective, our structural insights into SpXpr1 could inform the development of antifungal agents or crop improvement strategies targeting phosphate utilization^3,44^.

## Materials and methods

### SpXpr1 cloning, expression, and purification

The *Xpr1/Spx2* gene from *S. pombe* (UniProt: Q9UU86; PomBase: SPCC1827.07c) was PCR-amplified from WT genomic DNA, cloned, and inserted into a pEG BacMam expression vector containing an N-terminal Flag tag^45^. All the constructs were validated by Sanger sequencing prior to transfection. Expi293F cells (Gibco, Thermo Fisher Scientific) were cultured in SMM 293T-II medium (Sino Biological) at 37 °C with 5% CO₂ in a ZCZY-CS9 orbital shaker at 150 rpm (Zhichu Instrument). At a density of 2 × 10⁶ cells/mL, the cells were transfected with 2 mg of SpXpr1 plasmid DNA complexed with 5 mg of linear polyethylenimine (Yeasen) per liter of culture. After 48 h of expression, the cells were harvested by centrifugation, flash-frozen in liquid nitrogen, and stored at –80 °C.

For protein purification, frozen cell pellets were thawed and resuspended in lysis buffer (20 mM HEPES–NaOH (pH 7.4), 150 mM NaCl, 10 mM sodium phosphate, and 1% DDM/CHS [10:1, w/w; Anatrace]) supplemented with protease inhibitors (1 μM leupeptin, 1 μM pepstatin A, 1 μg/mL aprotinin, and 2 mM PMSF) and gently agitated for 2 h at 4 °C. After centrifugation at 13,000 rpm for 1 h at 4 °C, the clarified lysate was incubated with anti-DYKDDDDK G1 affinity resin (GenScript) and washed with 40 mL of wash buffer (20 mM HEPES–NaOH (pH 7.4), 150 mM NaCl, 10 mM sodium phosphate, 1 mM PMSF, and 0.01% GDN [Anatrace]). FLAG-tagged SpXpr1 was eluted with 10 mL of elution buffer containing 400 μg/mL FLAG peptide (GenScript) under the same detergent conditions. The eluate was concentrated using a 50 kDa MWCO Amicon Ultra-4 centrifugal filter (Millipore) and further purified by size-exclusion chromatography on a Superose 6 10/300 column (GE Healthcare) equilibrated with SEC buffer (20 mM HEPES–NaOH (pH 7.4), 150 mM NaCl, 10 mM sodium phosphate, 1 mM PMSF, 0.01% GDN). The peak fractions were pooled and concentrated for cryo-EM analysis. For purification of InsP6-bound SpXpr1, 1 mM InsP6 (Sigma-Aldrich #P8810) was included in all buffers throughout the purification process.

### Cryo-EM sample preparation and data collection

Concentrated protein (with or without 1 mM InsP6) was applied to glow-discharged Quantifoil Au R1.2/1.3 holey carbon grids and flash-frozen in liquid ethane cooled by liquid nitrogen using a Vitrobot (Mark IV, Thermo Fisher Scientific). The grids were then loaded onto a 300 kV Titan Krios equipped with a K3 Summit detector (Gatan) and a GIF Quantum energy filter. Automated data collection was performed using EPU software (Thermo Fisher Scientific) in super-resolution mode at a nominal magnification of 81,000×, with the energy filter set to a slit width of 20 eV. Images were acquired with a defocus range of –1.5 to –2.0 μm using a total exposure time of 2.56 s (0.08 s per frame, 32 frames in total), corresponding to a total dose of ~50 e^−^/Å². Movie stacks were processed with MotionCor2^46^ for motion correction and 2× binning, yielding a final pixel size of 1.087 Å/pixel with dose weighting applied. Defocus values were determined using Gctf^47^.

### Cryo-EM data processing

The data processing workflow is summarized in Supplementary Fig. S5. For the SpXpr1 and SpXpr1^+lnsP6^ datasets, 3033 and 5126 micrographs, respectively, were collected. All micrographs were processed using CryoSPARC v4.6.2^48^, yielding 2,283,456 and 5,814,611 auto-picked particles for SpXpr1 and SpXpr1^+lnsP6^, respectively. After multiple rounds of 2D classification, 237,376 (SpXpr1) and 240,024 (SpXpr1^+lnsP6^) particles were selected for subsequent processing, which included ab initio reconstruction, non-uniform refinement, and iterative heterogeneous refinement. The final selected particles (210,108 for SpXpr1 and 226,844 for SpXpr1^+lnsP6^) were subjected to local CTF refinement and non-uniform refinement, yielding 3D reconstructions at overall resolutions of 2.90 and 2.87 Å, respectively.

### Model building and structure refinement

The AlphaFold-predicted model of SpXpr1 (AlphaFold DB ID: Q9UU86) was initially docked into the final cryo-EM density maps of both SpXpr1 and SpXpr1^+lnsP6^ using UCSF ChimeraX^49–51^. Manual model construction and adjustment were performed in Coot v0.9.8.1^52^, with careful attention given to the chemical properties of the amino acid residues. Owing to insufficient electron density, the N-terminal and C-terminal regions were only partially modeled; specifically, residues 39–64, 115–126, 210–212, and 645–682 of SpXpr1 and residues 38–61, 112–145, 210–211, and 641–682 of SpXpr1^+InsP6^ were excluded. Several potential lipid molecules were tentatively modeled into the available density for both structures.

Real-space refinement was performed using phenix.real_space_refine in PHENIX 1.20 with secondary structure and geometric restraints^53^. To prevent overfitting, we employed the gold-standard refinement approach by alternately refining against one of the two independent half-maps and validating it against the other. The final refinement statistics and map quality metrics are presented in Supplementary Table S1.

### Construction of the *HsXPR1*-knockout HEK293T cell line

The *HsXPR1*-knockout HEK293T cell line was generated using the Sleeping Beauty transposon system coupled with CRISPR-Cas9^54–56^. Briefly, HEK293T cells were seeded at a density of 1 × 10⁶ cells per 10-cm dish. The cells were co-transfected using PEI with 10 µg of a donor plasmid encoding Cas9 targeting the human *XPR1* gene and a puromycin resistance gene, along with 0.2 µg of the SB100 transposase plasmid in 1 mL of Opti-MEM medium. The transfection medium was replaced with fresh complete culture medium 6 h post-transfection. Puromycin selection (final concentration of 10 µg/mL from a 10 mg/mL stock) was initiated approximately 48 h after transfection. Resistant cell pools were expanded and maintained under puromycin pressure. During this period, the cells were passaged at a 1:5 ratio upon reaching confluence. The selection process was continued for 2–3 passages until no cell death was observed, indicating complete elimination of non-transfected cells. Successful knockout was subsequently confirmed by genomic PCR followed by Sanger sequencing of the targeted locus to identify indel mutations and by western blotting to verify the absence of HsXPR1 protein expression.

### Phosphate efflux assay with SpXpr1 and its mutants

The phosphate efflux assay was adapted from established protocols with minor modifications^13,57^. Briefly, *HsXPR1*-knockout HEK293T cells were seeded in 12-well plates at 3 × 10⁵ cells per well in DMEM (Bio-channel) supplemented with 10% FBS (Cellmax) and cultured at 37 °C with 5% CO₂. Twenty-four hours later, the cells were transfected with 1 μg of plasmid DNA complexed with 2 μg of linear polyethylenimine (Yeasen) per well. At 24 h posttransfection, the cells were washed three times with phosphate-free DMEM (Gibco) and incubated in this same medium for 1 h at 37 °C. Conditioned media were collected, and extracellular phosphate concentrations were quantified using a Malachite Green Phosphate Assay Kit (MAK307, Sigma). For data normalization, the cells were lysed with 300 μL of RIPA buffer (Beyotime) and incubated for 10 min at room temperature. The expression levels of SpXpr1 and its mutants were visualized and quantified via western blotting, while total protein content was determined using a BCA assay (Beyotime) in 96-well plates. Phosphate export activity was calculated relative to the expression levels of specific proteins or total cellular proteins as needed. All experiments included triplicate wells per condition and were independently repeated at least three times on different days.

### Immunofluorescence staining

To verify plasma membrane localization, HEK293T cells expressing FLAG-tagged GFP (control), FLAG-tagged SpXpr1^WT^, and SpXpr1 mutants were analyzed by a Nikon NSPARC super-resolution microscope. Cells were seeded on coverslips (Biosharp) in 12-well plates prior to transfection. At 24 h posttransfection, the coverslips were transferred to fresh plates and washed with 1× PBS. Cells were fixed with 4% paraformaldehyde (PFA; Coolaber) for 15 min at room temperature, followed by three 5-min washes with PBS. Permeabilization was performed using 0.3% Triton X-100 in PBS for 30 min, and the cells were subsequently washed three times with PBS. Non-specific binding was blocked by incubation with 3% BSA in PBS containing 0.1% Triton X-100 for 30 min at room temperature. Primary antibody incubation was carried out using an anti-DYKDDDDK monoclonal antibody (Proteintech; 1:1000 in blocking buffer) for 2 h at room temperature. After three PBS washes, the cells were incubated with an AF647-conjugated goat anti-mouse IgG (H + L) secondary antibody (Beyotime; 1:200 in blocking buffer) for 1 h at room temperature. Following final washes with PBS, the coverslips were mounted using DAPI-containing antifade medium (Beyotime), sealed with nail polish, and imaged on a Nikon NSPARC confocal system.

### Western blot analysis

Protein samples extracted with RIPA lysis buffer were denatured in SDS‒PAGE loading buffer (Beyotime) and separated via a 4%–20% gradient SurePAGE gels (GenScript) at 140 V for 60 min. The proteins were then transferred to PVDF membranes using the eBlot™ L1 rapid wet blotting system (GenScript). The membranes were blocked with 5% non-fat dry milk in TBST for 1 h at room temperature, followed by five 5-min washes with TBST. Immunoblotting was performed using an anti-DYKDDDDK monoclonal antibody (Proteintech; 1:5000 dilution in antibody buffer) or an anti-XPR1 monoclonal antibody (GeneTex, 1:1000 dilution in antibody buffer) as the primary antibody, followed by a goat anti-mouse/rabbit IgG HRP-conjugated secondary antibody (CWBIO; 1:5000 in TBST) with five subsequent TBST washes. The protein bands were visualized using BeyoECL Moon chemiluminescent substrate (Beyotime) and imaged on an Amersham Imager 680 system (Cytiva).

### Electrophysiological assay

For the electrophysiological experiments, HEK293T cells were cultured in Dulbecco’s modified Eagle’s medium (DMEM) (Corning, USA) supplemented with 1% (w/v) penicillin/streptomycin (Gibco, USA), 1% (w/v) glutamate (Gibco), and 10% (v/v) fetal bovine serum (FBS) (PAN-Biotech GmbH, Germany) at 37 °C in a humidified atmosphere containing 5% CO_2_ and 95% air, following established protocols^58^. WT SpXpr1 and its mutants in the pEG BacMam vector were transfected into HEK293T cells using the lipid transfection reagent Lipo293TM (Beyotime). The transfected cells were cultured for 24 h before the electrophysiological experiments were conducted.

All electrophysiological recordings were conducted with HEK293T cells at room temperature (23 ± 2 °C) using an Axon 200B amplifier combined with a Digitata1550 digitizer (Axon Instruments, USA), as outlined in previous studies^58^. Data acquisition and analysis were carried out using pCLAMP 10.7 software (Axon Instruments). For Pi current detection, the bath solution contained 150 mM N-methyl-D-glucamine (NMDG), 100 mM H_3_PO_4_, and 10 mM HEPES (pH 7.4), and the pipette solution contained 150 mM NMDG, 100 mM H_3_PO_4_, 10 mM HEPES, and 10 mM EGTA. An additional 1 mM InsP6 was added to the pipette solution for experiments involving InsP6. The experimental setup utilized a step-voltage protocol ranging from −180 mV to +100 mV with a +20 mV step over 1–3 min after the whole-cell configuration was achieved^59^.

### Inclusion and ethics statement

All the collaborators in this study meet the authorship criteria required by Nature Portfolio journals and have been duly included as authors. Roles and responsibilities were agreed upon by all the collaborators prior to the commencement of the research. This study does not result in stigmatization, incrimination, discrimination, or any other personal risk to participants. Additionally, the research does not pose health, safety, security, or other risks to the researchers involved. We have discussed benefit-sharing measures and ensured that local and regional research relevant to this study is appropriately cited.

## Supplementary information

Supplementary Information

## Data Availability

Atomic coordinates and EM maps of SpXpr1 (PDB: 9W0X; Electron Microscopy Data Bank (EMDB): EMD-65520) and SpXpr1^+InsP6^ (PDB: 9W1B; EMDB: EMD-65525) have been deposited in the Protein Data Bank (http://www.rcsb.org) and the Electron Microscopy Data Bank (https://www.ebi.ac.uk/pdbe/emdb/), respectively.
