# Supplementary material for: Structural insights into the gating mechanism of the fission yeast phosphate exporter SpXpr1
Source: Cell Discov. 2026 Apr 15;12:27. doi: 10.1038/s41421-026-00883-8 (PMC13083964; doi:10.1038/s41421-026-00883-8)
Supplement: Supplementary file 1 — Supplementary Information [file 41421_2026_883_MOESM1_ESM.pdf]

Structural insights into the gating mechanism of a fission yeast phosphate exporter SpXpr1

Hui Yang[1,2,3,4,6], Yuechan Wang[1,2,3,4,6], Chenxi Yue[5,6], Xinran Li[2,3,4], Yifei Wang[2,3,4], Ye Yu[5,7], and Huaizong Shen[2,3,4,7]

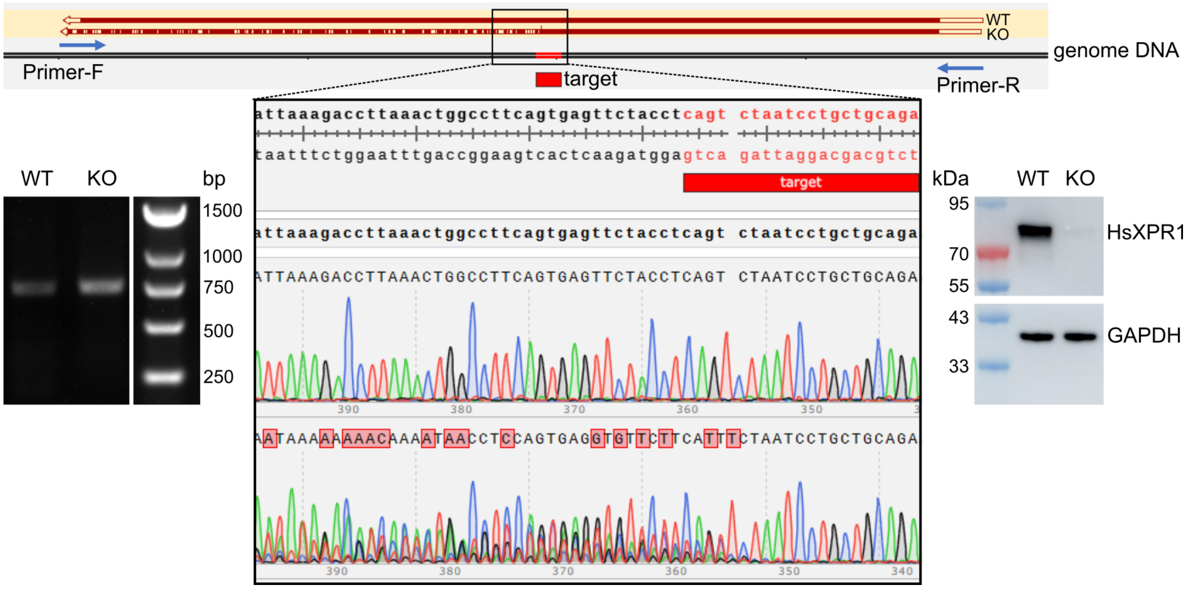

Supplementary Fig. 1 | Validation of the constructed *HsXPR1*-knockout 293T cell line. The target genomic locus of *HsXPR1* was PCR-amplified from wild-type (WT) and knockout (KO) cell lines using specific primers (left) and analyzed by Sanger sequencing (middle). Sequencing chromatograms revealed frameshift-inducing indel mutations at the target site in the KO clone compared to the WT control. Western blot analysis confirmed the absence of HsXPR1 protein expression in the KO cell line (right). GAPDH served as the loading control.

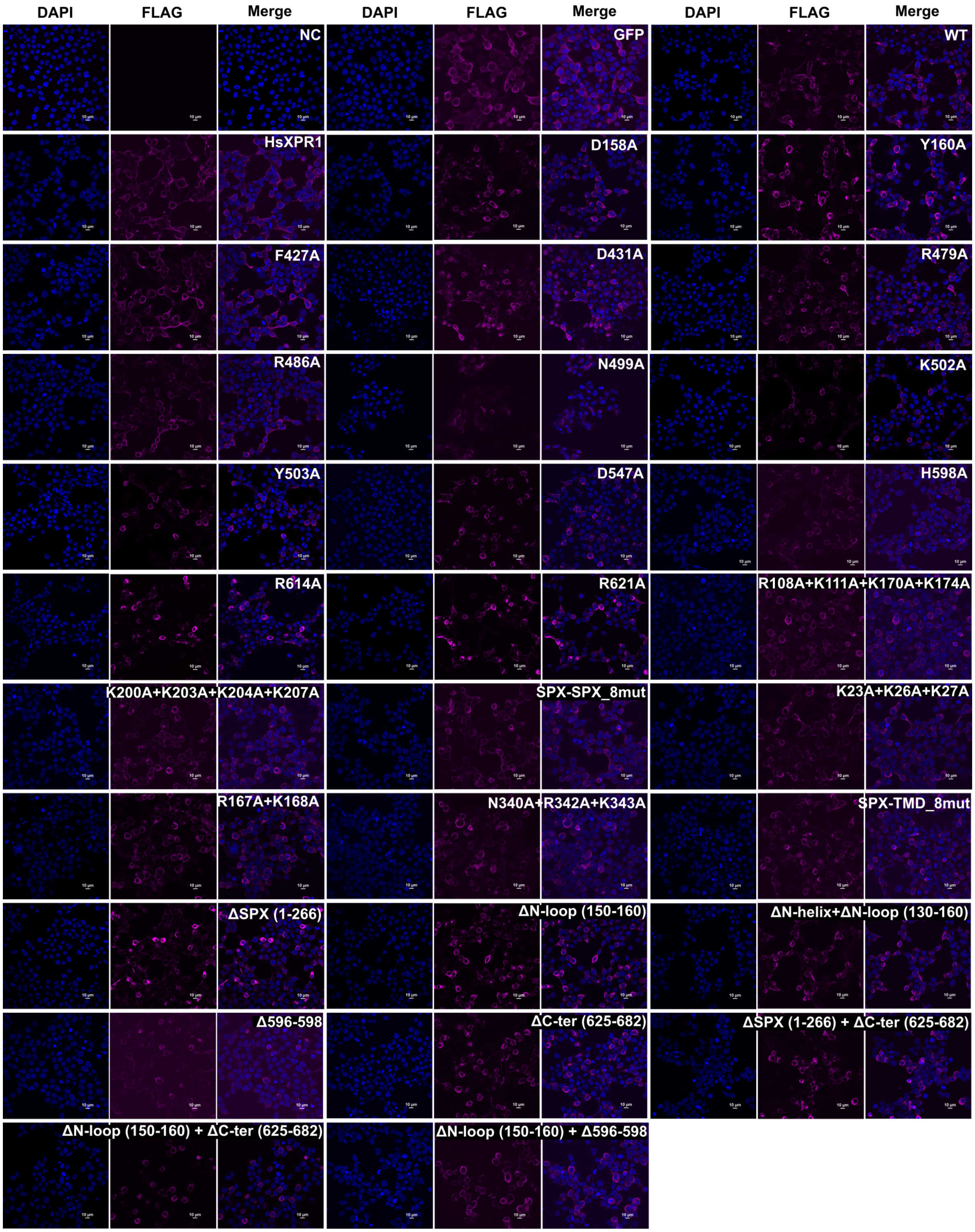

Supplementary Fig. 2 | Cell surface expression profiles of SpXpr1 and mutants in *HsXPR1*-KO 293T cells. The expression and localization of SpXpr1 and mutants were

analyzed using a Nikon NSPARC super-resolution microscope. The first column displays DAPI staining to mark nuclei, while the second column presents the expression profiles of FLAG-tagged SpXpr1 and its mutants. Both fluorescence signals were acquired and merged for visualization. NC, negative control.

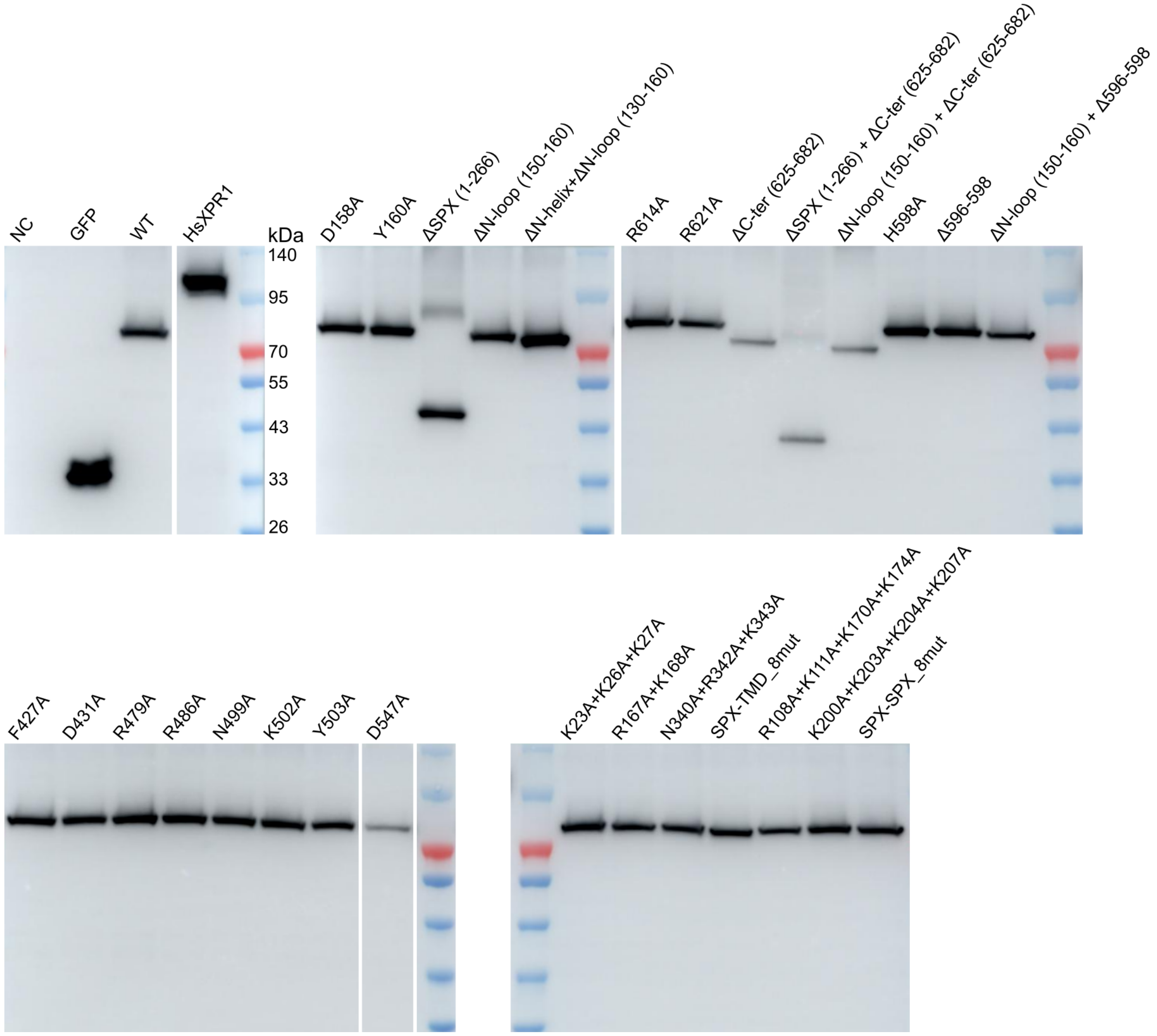

Supplementary Fig. 3 | Representative expression profiles of SpXpr1 and mutants in *HsXPR1*-KO 293T cells. The expression levels of FLAG-tagged SpXpr1 and its mutants, used in the phosphate export assays, were verified by Western blot analysis. To minimize differences in band exposure during WB imaging, 1 µL of GFP samples and 20 µL of other samples were loaded onto the gel. NC, negative control.

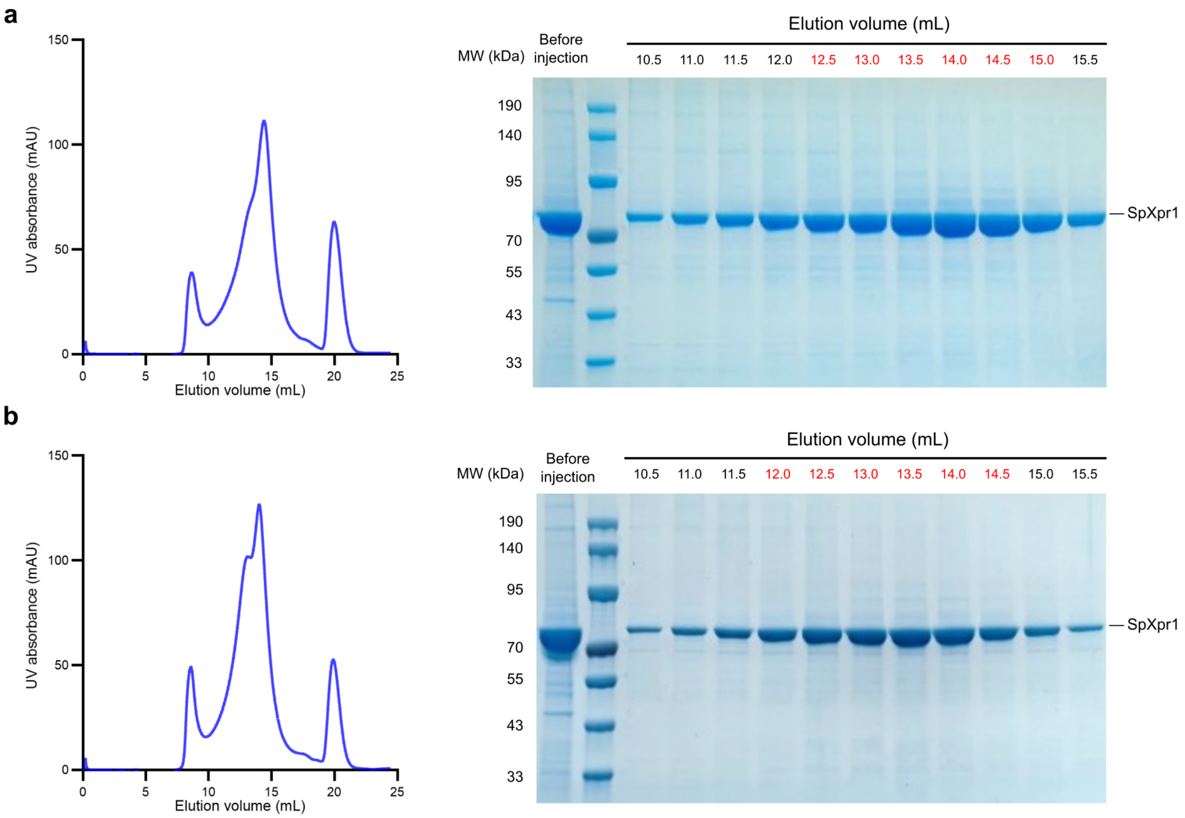

Supplementary Fig. 4 | Size-exclusion chromatography (SEC) profiles of SpXpr1. (a, b) Size exclusion chromatography (SEC) profile and SDS-PAGE gel for preparations of apo (**a**) and InsP6-bound (**b**) SpXpr1 are displayed. The peak fractions for the final sample preparations are colored in red on the Coomassie blue-stained SDS-PAGE gels.

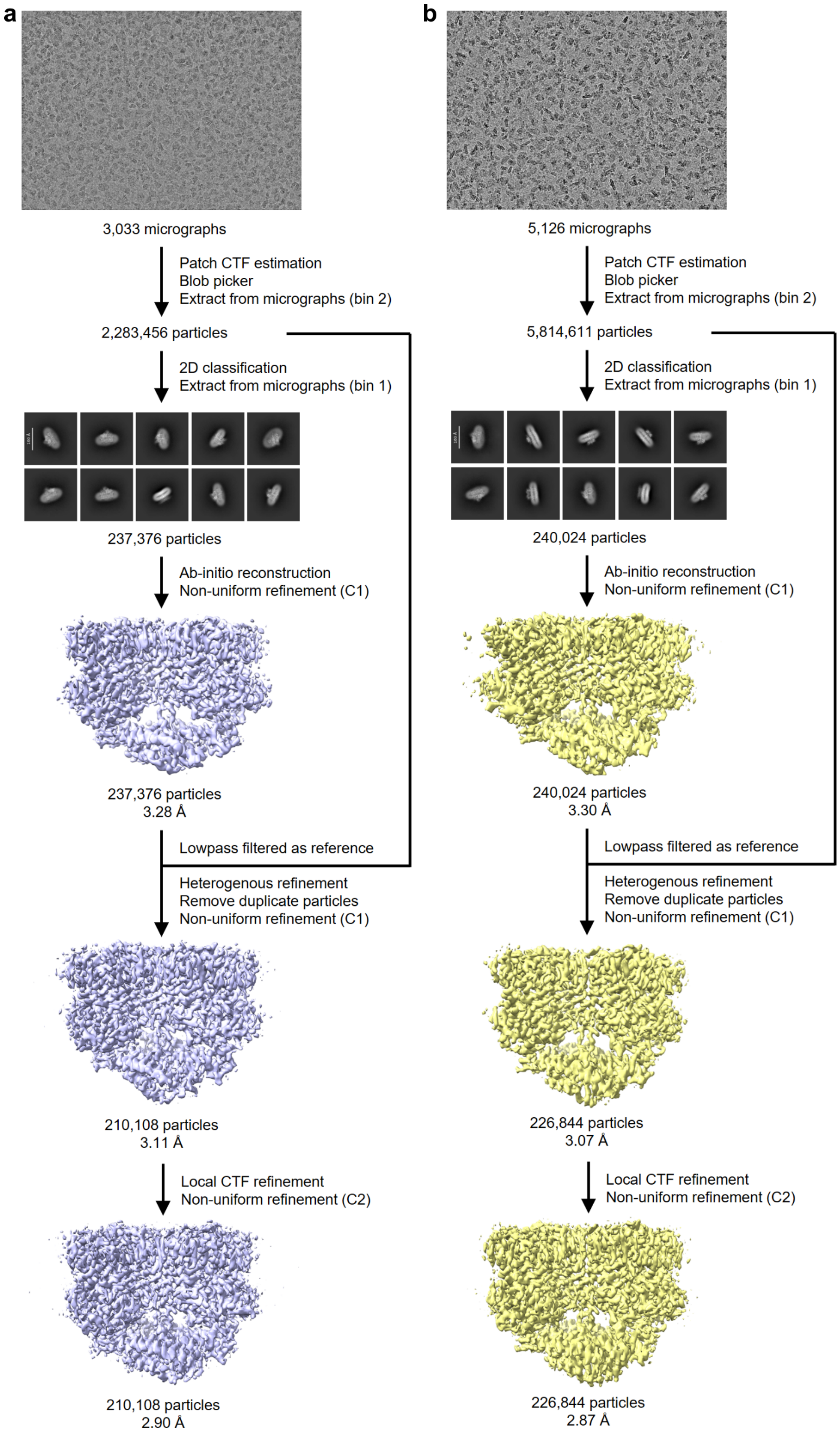
a
3,033 micrographs
Patch CTF estimation
Blob picker
Extract from micrographs (bin 2)
2,283,456 particles
2D classification
Extract from micrographs (bin 1)
237,376 particles
Ab-initio reconstruction
Non-uniform refinement (C1)
237,376 particles
3.28 Å
Lowpass filtered as reference
Heterogenous refinement
Remove duplicate particles
Non-uniform refinement (C1)
210,108 particles
3.11 Å
Local CTF refinement
Non-uniform refinement (C2)
210,108 particles
2.90 Å
b
5,126 micrographs
Patch CTF estimation
Blob picker
Extract from micrographs (bin 2)
5,814,611 particles
2D classification
Extract from micrographs (bin 1)
240,024 particles
Ab-initio reconstruction
Non-uniform refinement (C1)
240,024 particles
3.30 Å
Lowpass filtered as reference
Heterogenous refinement
Remove duplicate particles
Non-uniform refinement (C1)
226,844 particles
3.07 Å
Local CTF refinement
Non-uniform refinement (C2)
226,844 particles
2.87 Å

Supplementary Fig. 5 | Cryo-EM data processing flowcharts of SpXpr1. (a, b) Cryo-EM data processing flowcharts for apo (**a**) and InsP6-bound (**b**) SpXpr1 are illustrated in detail. Representative 2D and 3D results are illustrated with available resolutions for 3D reconstructions indicated. Data processing was performed using CryoSPARC[48].

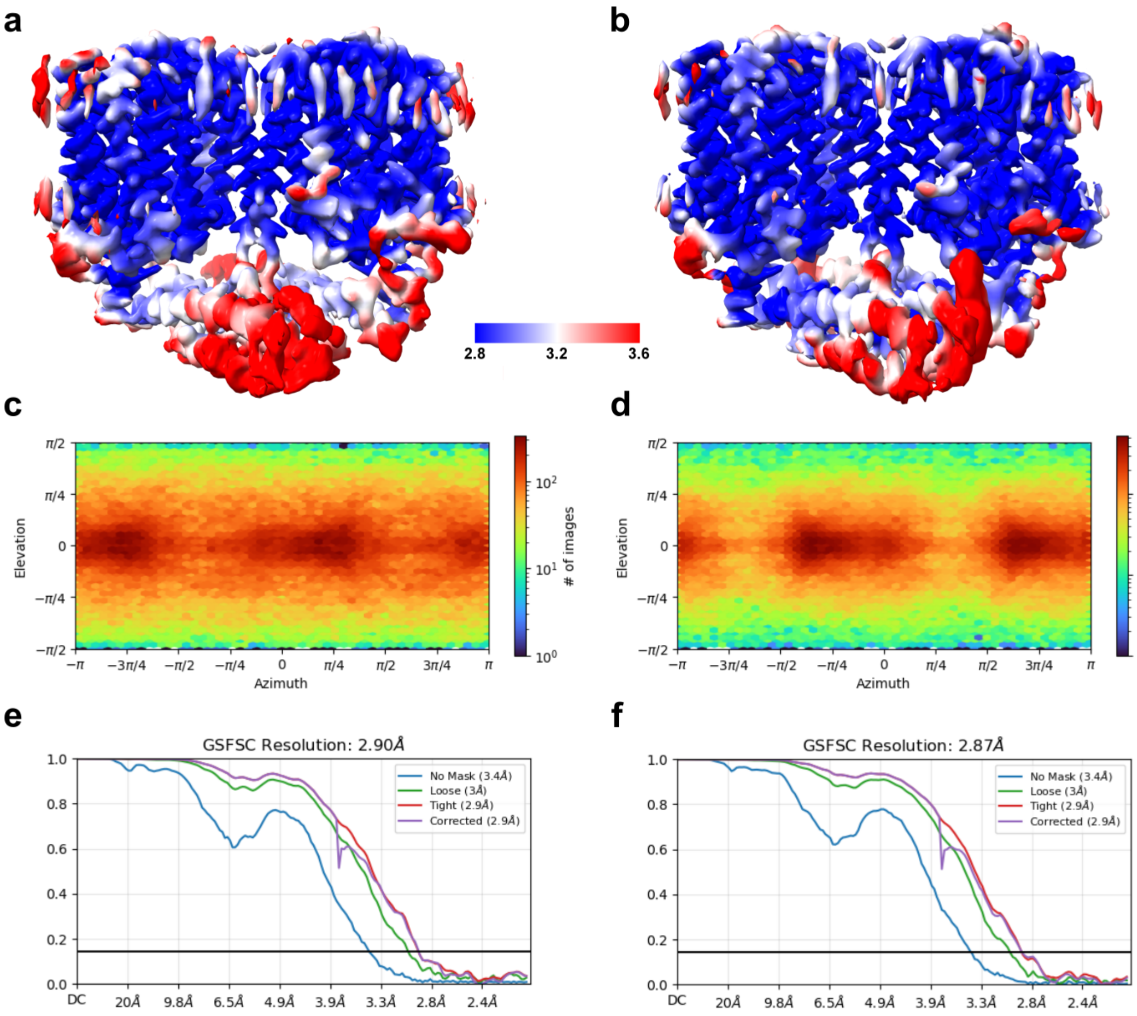

Supplementary Fig. 6 | Final reconstruction of cryo-EM maps for SpXpr1. (a, b) Local resolution distribution maps for apo (**a**) and InsP6-bound (**b**) SpXpr1. **(c, d)** Particle angular distribution plots calculated in CryoSPARC[48] for the final reconstruction of apo (**c**) and InsP6-bound (**d**) SpXpr1. **(e, f)** Fourier-shell correlation (FSC) analysis of apo (**e**) and InsP6-bound (**f**) SpXpr1 generated by non-uniform refinement function in cryoSPARC.

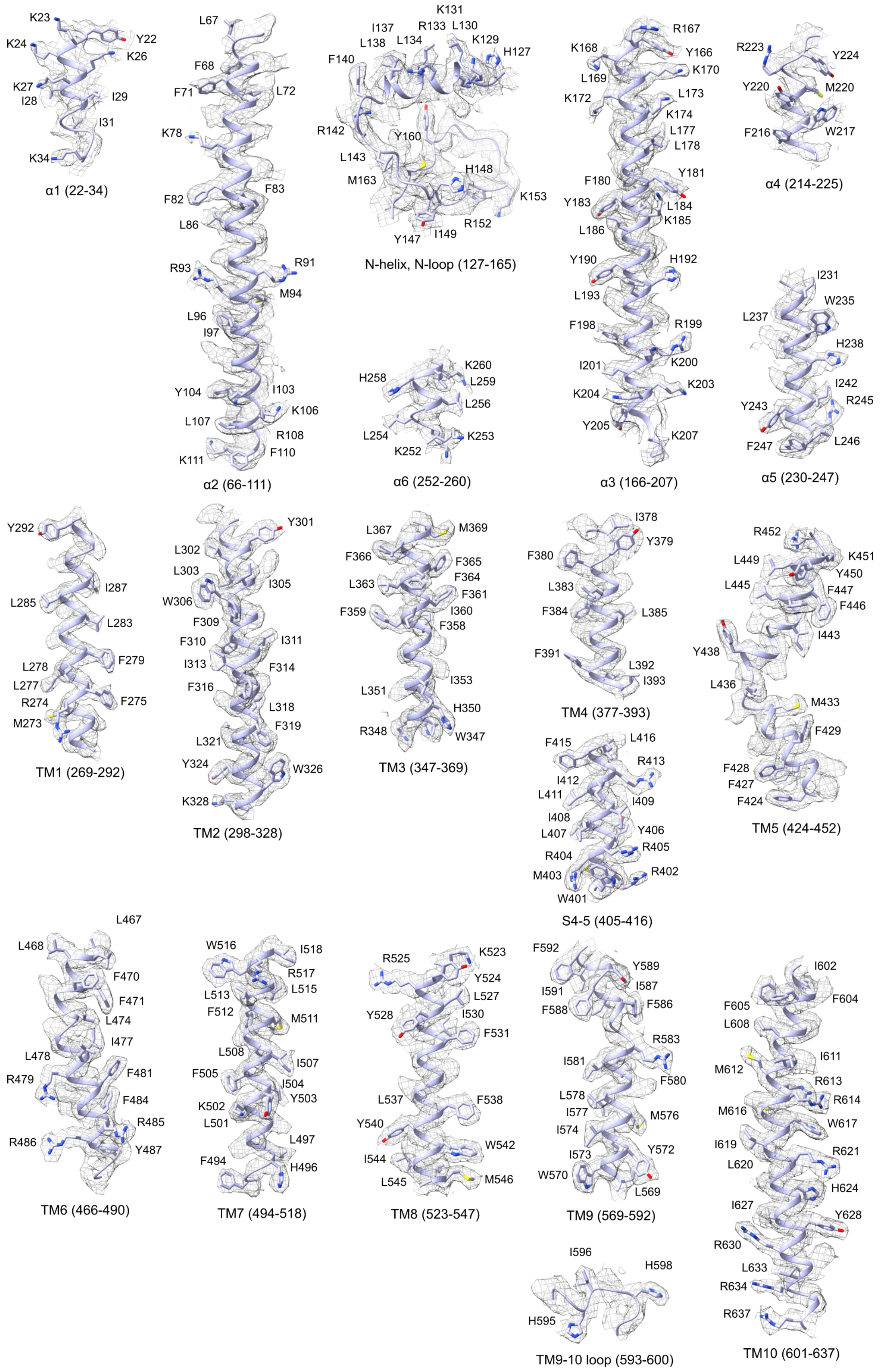
K23
Y22
K24
K26
K27
I28
I29
I31
K34
α1 (22-34)
L67
F68
F71
L72
K78
F82
F83
L86
R93
R91
M94
L96
I97
Y104
I103
K106
L107
R108
K111
F110
α2 (66-111)
K131
I137
R133
L130
L138
L134
K129
F140
H127
R142
Y160
L143
H148
M163
K153
R152
Y147
I149
N-helix, N-loop (127-165)
H258
K260
L259
L256
L254
K253
K252
α6 (252-260)
R167
K168
Y166
K170
L169
K172
L173
K174
L177
L178
Y181
F180
Y183
L184
K185
L186
Y190
H192
L193
F198
R199
I201
K200
K204
K203
Y205
K207
α3 (166-207)
R223
Y224
Y220
M220
F216
W217
α4 (214-225)
I231
W235
L237
H238
I242
Y243
R245
F247
L246
α5 (230-247)
Y292
I287
L285
L283
L278
F279
L277
R274
F275
M273
TM1 (269-292)
Y301
L302
L303
I305
W306
F309
F310
I311
I313
F314
F316
L318
F319
L321
Y324
W326
K328
TM2 (298-328)
L367
M369
F366
F365
F364
L363
F361
F359
I360
F358
I353
L351
H350
R348
W347
TM3 (347-369)
I378
Y379
F380
L383
F384
L385
F391
L392
I393
TM4 (377-393)
F415
L416
R413
I412
L411
I409
I408
Y406
L407
R405
R404
M403
R402
W401
S4-5 (405-416)
R452
K451
L449
Y450
L445
F447
F446
I443
Y438
L436
M433
F429
F428
F427
F424
TM5 (424-452)
L467
L468
F470
F471
L474
I477
L478
F481
R479
F484
R485
R486
Y487
TM6 (466-490)
W516
I518
R517
L515
L513
F512
M511
L508
I507
F505
I504
Y503
K502
L501
L497
F494
H496
TM7 (494-518)
K523
R525
Y524
L527
Y528
I530
F531
L537
F538
Y540
W542
I544
L545
M546
TM8 (523-547)
F592
Y589
I587
I591
F586
F588
R583
I581
F580
L578
I577
M576
I574
I573
Y572
W570
L569
TM9 (569-592)
I596
H598
H595
TM9-10 loop (593-600)
I602
F604
F605
L608
I611
M612
R613
R614
M616
W617
I619
R621
L620
H624
I627
Y628
R630
L633
R634
R637
TM10 (601-637)

51

Supplementary Fig. 7 | Cryo-EM densities of apo SpXpr1. The electron microscopy (EM) densities of apo SpXpr1 structure were visualized using UCSF ChimeraX[51]. Residues with large side chains are labeled.

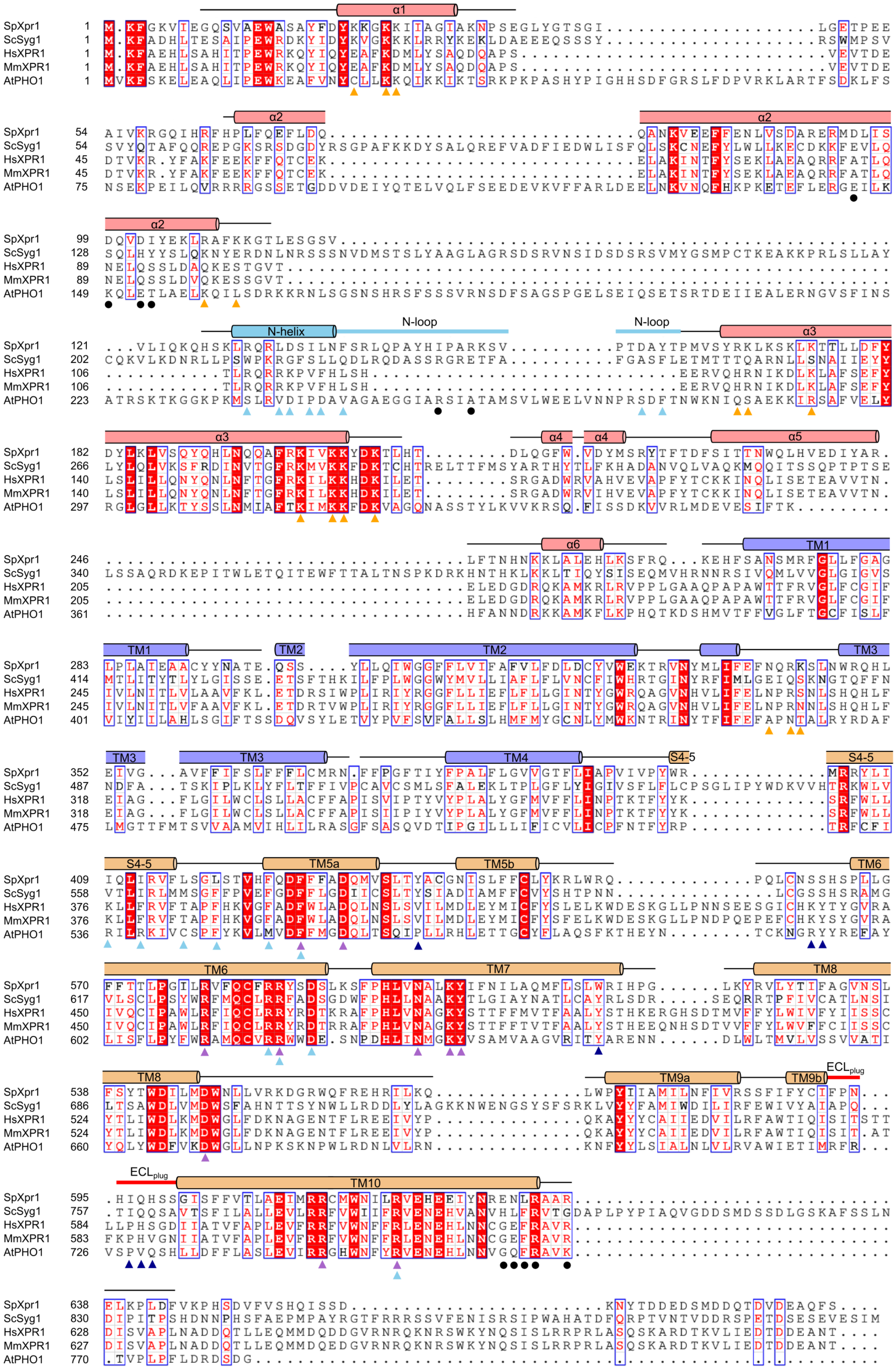
α1
α2
α3
α4
α5
α6
N-helix
N-loop
TM1
TM2
TM3
TM4
S4-5
TM5a
TM5b
TM6
TM7
TM8
TM9a
TM9b
TM10
ECLplug
SpXpr1
ScSyg1
HsXPR1
MmXPR1
AtPHO1

Supplementary Fig. 8 | Sequence alignment of XPR1 homologs. Sequence alignments were performed for XPR1 homologs from *Schizosaccharomyces pombe* (Sp), *Homo sapiens* (Hs), and *Mus musculus* (Mm), Syg1 from *Saccharomyces cerevisiae* (Sc), and PHO1 from *Arabidopsis thaliana* (At). Secondary structure elements are indicated above the alignments. Functionally important residues are marked with colored symbols: light purple triangles (phosphate-binding), sky blue triangles (N-helix/N-loop interactions), dark blue triangles (ECL_{plug} interactions), orange triangles (InsP6-binding), and black circles (C-terminal interactions).

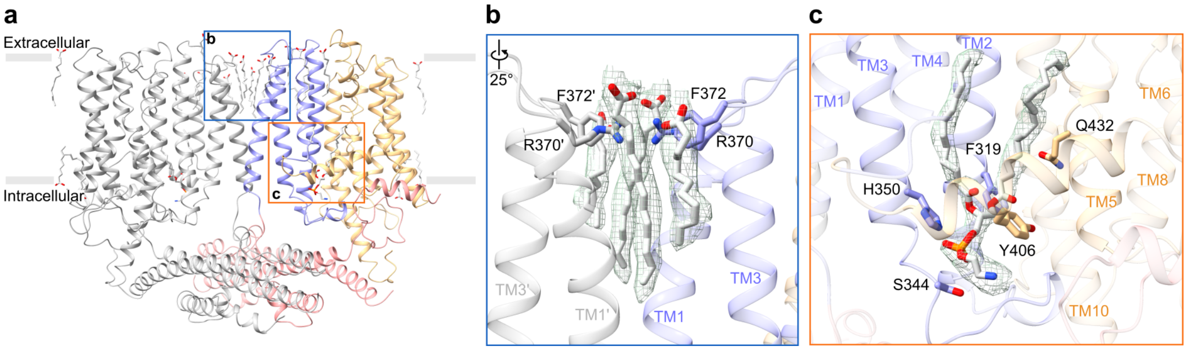

Supplementary Fig. 9 | Lipids disclosed in the structure. (a) Overall structure of the apo SpXpr1 dimer, with the left protomer shown in grey. Lipid molecules are presented as stick models. **(b, c)** Detailed views of the dimer interface on the extracellular (**b**) and intracellular (**c**) sides. Key residues involved in the interface are depicted as stick models.

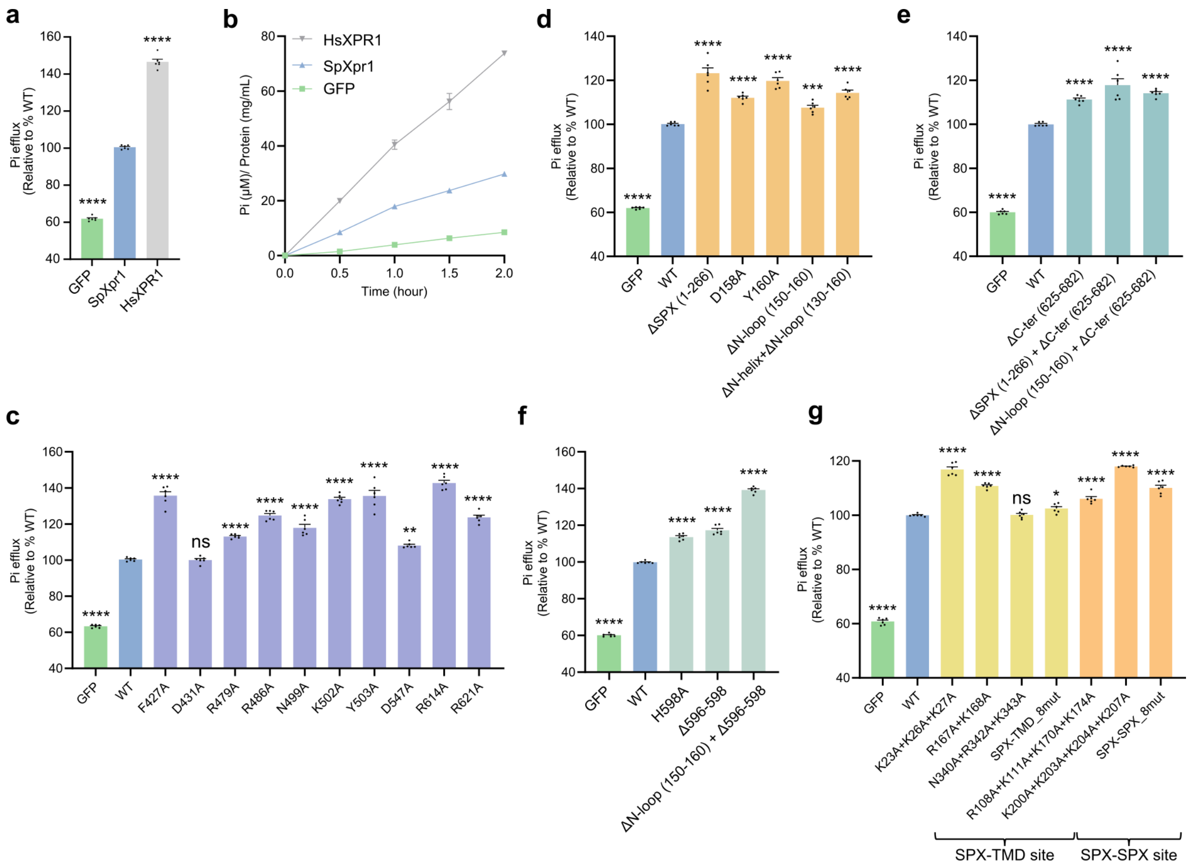

Supplementary Fig. 10 | Phosphate export activities normalized to total cellular protein levels in *HsXPR1*-KO 293T cells. (a,b) Phosphate export activities of SpXpr1, HsXPR1, and GFP in a direct comparison **(a)** and over time **(b)**. **(c-g)** Phosphate export activities of wild-type SpXpr1 and mutants targeting key functional sites: phosphate coordination **(c)**, N-loop stabilization **(d)**, C-terminal interaction **(e)**, ECL_{plug} interaction **(f)**, and InsP6 binding **(g)**. The efflux activity was normalized to total protein concentration (BCA assay) and then compared to that of WT (set as 100%). Cells expressing GFP served as controls. Results are presented as mean ± SEM, with n = 6 from three independent experiments. Statistical analysis was performed using one-way ANOVA with Dunnett's test against the WT group. Significance levels: *P ≤ 0.05, **P ≤ 0.01, ***P ≤ 0.001, ****P≤0.0001; ns, not significant.

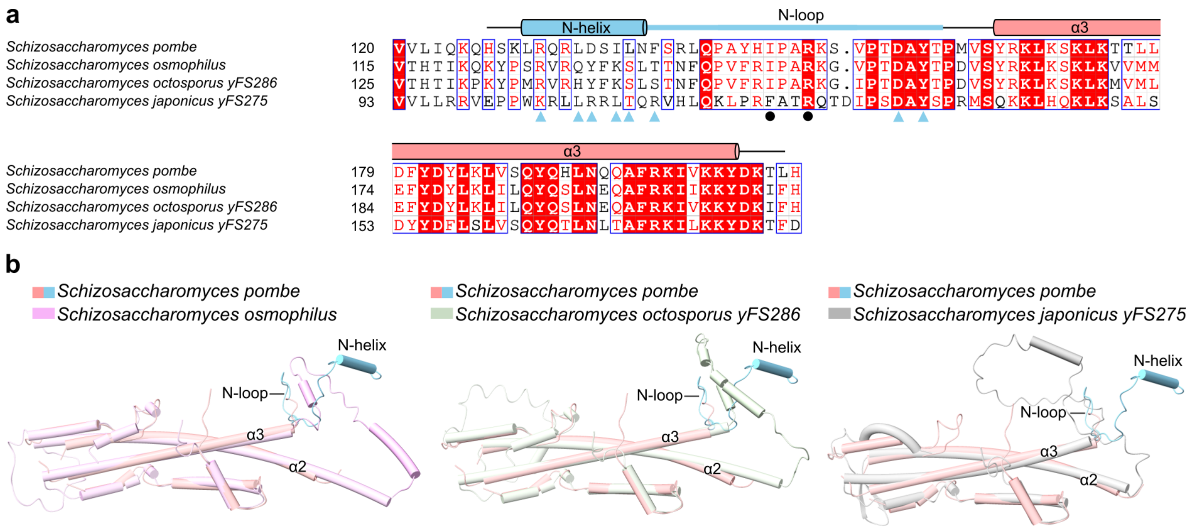

Supplementary Fig. 11 | Conservation of the N-helix/N-loop module in fission yeast Xpr1 homologs. (a) Sequence alignment of the N-terminal regions from four *Schizosaccharomyces* species (*S. pombe*, *S. osmophilus*, *S. octosporus yFS286*, and *S. japonicus yFS275*). Functionally important residues are marked with colored symbols: sky blue triangles (N-helix/N-loop interactions) and black circles (C-terminal interactions). **(b)** Structural superposition of the SPX domains. The experimental structure of SpXpr1 was aligned with the AlphaFold-predicted models of the other three homologs[50], revealing a conserved architectural feature—the N-helix and N-loop module between α2 and α3—across all species.

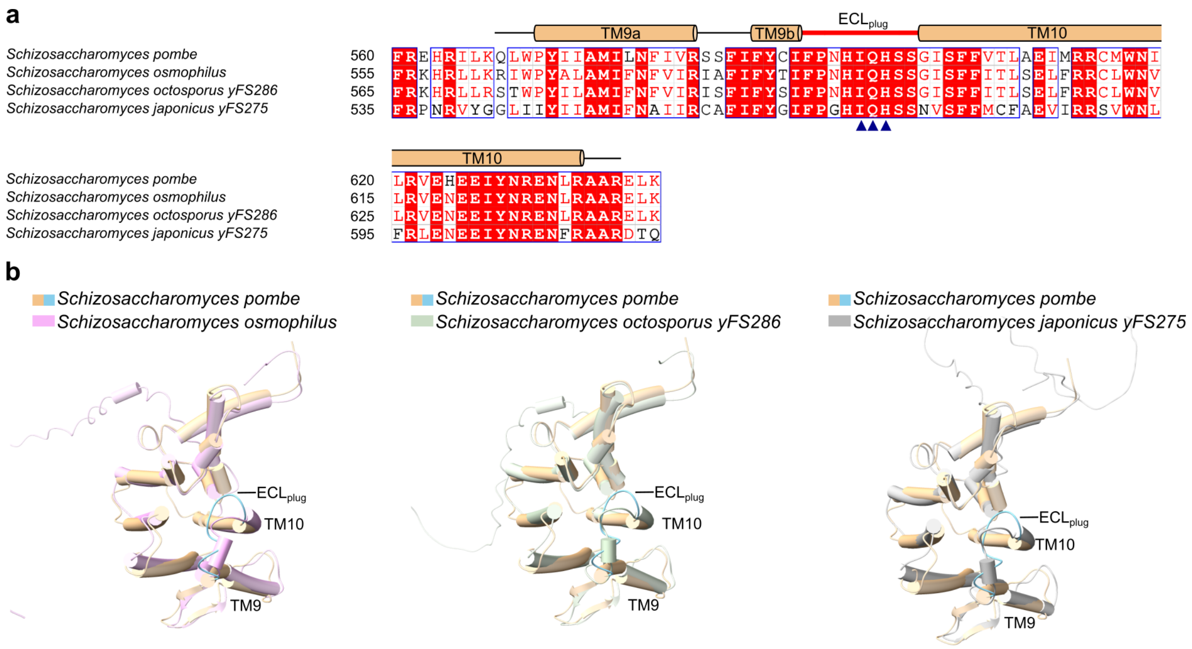

Supplementary Fig. 12 | Conservation of the ECL$_{plug}$ in fission yeast Xpr1 homologs. **(a)** Sequence alignment of the C-terminal regions from four *Schizosaccharomyces* species (*S. pombe, S. osmophilus*, *S. octosporus yFS286*, and *S. japonicus yFS275*). Residues critical for ECL$_{plug}$ interactions are indicated by dark blue triangles. **(b)** Structural superposition of the transmembrane domains. The experimental structure of SpXpr1 was aligned with the AlphaFold-predicted models of the other three homologs[50], revealing a conserved architectural feature—the ECL$_{plug}$ between TM9 and TM10—across all species.

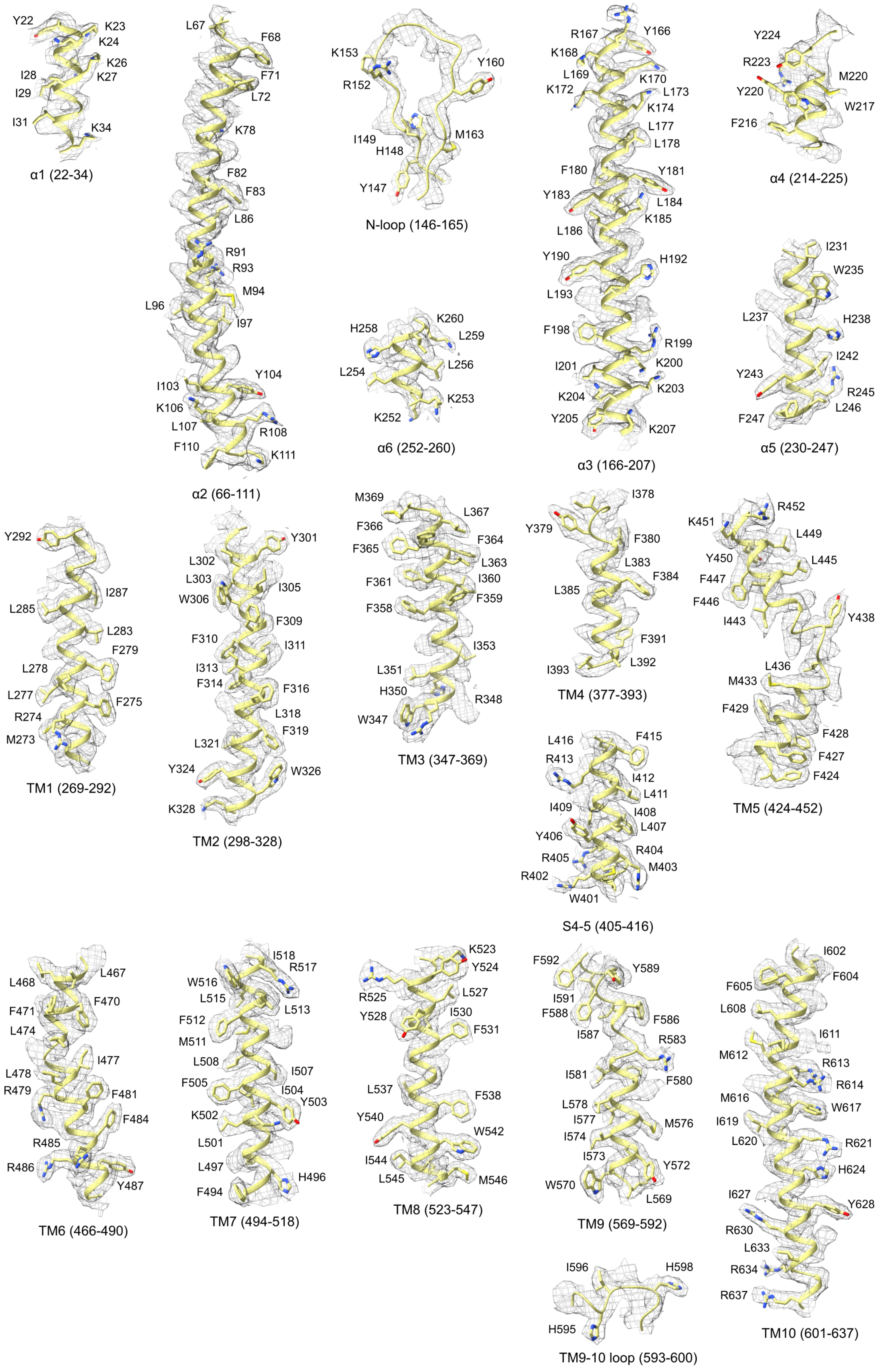
Y22
K23
K24
K26
I28
K27
I29
I31
K34
α1 (22-34)
L67
F68
F71
L72
K78
F82
F83
L86
R91
R93
M94
L96
I97
Y104
I103
K106
L107
R108
F110
K111
α2 (66-111)
K153
Y160
R152
I149
M163
H148
Y147
N-loop (146-165)
K260
H258
L259
L254
L256
K253
K252
α6 (252-260)
R167
Y166
K168
L169
K170
K172
L173
K174
L177
L178
F180
Y181
Y183
L184
K185
L186
Y190
H192
L193
F198
R199
K200
I201
K203
K204
Y205
K207
α3 (166-207)
Y224
R223
M220
Y220
W217
F216
α4 (214-225)
I231
W235
L237
H238
I242
Y243
R245
L246
F247
α5 (230-247)
Y292
I287
L285
L283
F279
L278
L277
F275
R274
M273
TM1 (269-292)
Y301
L302
L303
I305
W306
F309
F310
I311
I313
F314
F316
L318
F319
L321
Y324
W326
K328
TM2 (298-328)
M369
L367
F366
F364
F365
L363
I360
F361
F359
F358
I353
L351
H350
R348
W347
TM3 (347-369)
I378
Y379
F380
L383
F384
L385
F391
L392
I393
TM4 (377-393)
L416
F415
R413
I412
L411
I409
I408
L407
Y406
R404
R405
M403
R402
W401
S4-5 (405-416)
R452
K451
L449
Y450
L445
F447
F446
Y438
I443
L436
M433
F429
F428
F427
F424
TM5 (424-452)
L467
L468
F470
F471
L474
I477
L478
R479
F481
F484
R485
R486
Y487
TM6 (466-490)
I518
R517
W516
L515
L513
F512
M511
L508
I507
F505
I504
Y503
K502
L501
L497
H496
F494
TM7 (494-518)
K523
Y524
R525
L527
Y528
I530
F531
L537
F538
Y540
W542
I544
L545
M546
TM8 (523-547)
F592
Y589
I591
F588
F586
I587
R583
I581
F580
L578
I577
M576
I574
I573
Y572
W570
L569
TM9 (569-592)
I596
H598
H595
TM9-10 loop (593-600)
I602
F604
F605
L608
I611
M612
R613
R614
M616
W617
I619
L620
R621
H624
I627
Y628
R630
L633
R634
R637
TM10 (601-637)

Supplementary Fig. 13 | Cryo-EM densities of SpXpr1^{+InsP6}. The electron microscopy (EM) densities of InsP6-bound SpXpr1 (SpXpr1^{+InsP6}) structure were visualized using UCSF ChimeraX[51]. Residues with large side chains are labeled.

Supplementary Table 1 | Statistics for data collection and structural refinement.

**Data collection**	SpXpr1	$SpXpr1^{+InsP6}$
EM equipment	Titan Krios (Thermo Fisher Scientific Inc.)	
Voltage (kV)	300	
Detector	Gatan K3 Summit	
Energy filter	Gatan GIF Quantum, 20 eV slit	
Pixel size (Å)	1.087	
Electron dose (e^-/Å^2)	50	
Defocus range (μm)	-1.5 ~ -2.0	
Number of collected movie stacks	3,033	5,126
**Reconstruction**		
Software	CryoSPARC v4	
Number of used particles	210,108	226,844
Symmetry	C2	C2
Overall resolution (Å)	2.90	2.87
Map resolution range (Å)	2.69 ~ 3.20	2.63 ~ 3.27
Map sharpening B-factor (Å^2)	-110.5	-108.8
**Refinement**		
Software	Phenix	
Cell dimensions		
a=b=c (Å)	391.32	
α=β=γ (˚)	90	
Model composition		
Protein residues	1,190	1,144
Side chains assigned	1,190	1,144
PO_4^{3-}	4	
POV	18	
8PE	2	
IHP	0	4
R.m.s deviations		
Bonds length (Å)	0.007	0.009
Bonds angle (˚)	0.927	1.005
Ramachandran plot statistics (%)		
Preferred	94.29	95.04
Allowed	5.03	4.79
Outlier	0.68	0.18
